# The Ameliorative Effects of Arctiin and Arctigenin on the Oxidative Injury of Lung Induced by Silica via TLR-4/NLRP3/TGF-*β* Signaling Pathway

**DOI:** 10.1155/2021/5598980

**Published:** 2021-07-17

**Authors:** Xueying Liu, Jian Wang, Peiyuan Dou, Xu Zhang, Xiaoku Ran, Linlin Liu, Deqiang Dou

**Affiliations:** ^1^College of Pharmacy, Liaoning University of Traditional Chinese Medicine, Dalian 116600, China; ^2^Department of Medicinal Chemistry, Shenyang Pharmaceutical University, Shenyang 110032, China

## Abstract

Silicosis remains one of the most serious diseases worldwide, with no effective drug for its treatment. Our research results have indicated that arctiin and arctigenin could increase the mitochondrial membrane potential, which in turn reduces the production of reactive oxygen species (ROS), blocks the polarization of macrophages, and inhibits the differentiation of myofibroblasts to reduce oxidative stress, inflammation, and fibrosis. Further, our study revealed that arctiin and arctigenin suppressed the activation of NLRP3 inflammasome through the TLR-4/Myd88/NF-*κ*B pathway and the silica-induced secretion of TNF-*α*, IL-1*β*, TGF-*β*, and *α*-SMA. Besides, the silica-induced increase in the levels of serum ceruloplasmin and HYP was also inhibited. Results of metabolomics indicated that arctiin and arctigenin could regulate the abnormal metabolic pathways associated with the development of silicosis, which involve pantothenate and CoA biosynthesis, cysteine and methionine metabolism, linoleic acid metabolism, and arginine and proline metabolism successively. Furthermore, the analysis of metabolomics, together with network topological analysis in different phases of silicosis, revealed that urine myristic acid, serum 4-hydroxyproline, and L-arginine could be regarded as diagnosis biomarkers in the early phase and formation of pulmonary fibrosis in the latter phases of silicosis. Arctiin and arctigenin could downregulate the increased levels of myristic acid in the early phase and serum 4-hydroxyproline in the latter phase of silicosis. Interestingly, the integration of TLR-4/NLRP3/TGF-*β* signaling and metabolomics verified the importance of macrophage polarization in the silicosis fibrosis process. To the best of our knowledge, this is the first study reporting that arctiin and arctigenin both can ameliorate silicosis effectively, and the former is a little stronger than its aglycone arctigenin because of its high oral bioavailability, low toxicity, and multimolecular active metabolites as determined by AdmetSAR and molecular docking analysis.

## 1. Introduction

Silicosis is an occupational disease characterized by chronic lung inflammation, progressive pulmonary fibrosis, and systemic immune dysfunction [1–3]. The innate and adaptive immune systems are regarded to play key regulatory roles in early phases of silicosis [4]. Previous studies have indicated that silica can damage macrophages, activate the innate immune system, and initiate an inflammatory response in the lungs. As silicosis progresses, fibrosis and inflammation occur together; oxygen- and nitrogen-derived free radicals play a major role in silica-induced lung injury and fibrosis [5]. Silicosis is usually ignored or misdiagnosed as other diseases because only inflammation occurs at its early phase. Presently, X-ray is the primary method for diagnosing silicosis, but it is not suitable for early diagnosis as the image shadows only show the fibrosis formation. Fibrosis can lead to overdeposition of extracellular matrix, and ultimately lead to structural reconstitution of lung tissue and even respiratory failure. The mortality rate after lung fibrosis generally accounts for 50% to 70% [6, 7]. It is well known that it is difficult to recover after fibrosis; thus, early diagnosis and treatment for silicosis are important.

Metabolomics can comprehensively reflect changes in the metabolites of organisms in different states. The measurement of metabolite levels and variations in biofluids can offer many insights into disease processes and response to therapeutic intervention [8]. Urine is one of the terminal products of organisms, and its collection is simple and noninvasive to the body. Urine metabolites can reflect metabolism disorders and provide insights into the responses of the body to physiological dynamic changes or disease processes [8–10]. In addition, the composition and content of urine metabolites can also reflect changes in the body's metabolic network under the influence of diseases and drugs, and these attributes are essential for addressing the challenges associated with biomarker discovery [8]. Network pharmacology, as a newly developed strategy, focuses on searching for the relationships between compounds and their potential targets, and can help elucidate the molecular mechanism of action in metabolomics [11]. Moreover, molecular docking could be used not only to screen the action target and mechanism of drugs but also to provide the probable binding ability of drugs to the target [12].

*Fructus Arctii*, the dried fruit of *Arctium lappa* L., has the function of clearing away heat and nourishing the lung as recorded in China's Pharmacopoeia [13]. Arctiin is its main active component and accounting for over 5% of *Fructus Arctii* as stipulated in Chinese Pharmacopoeia [13]. Research indicated that arctigenin has the function of antioxidation, anti-inflammation [14, 15], antitumor [16], and neuron-protective activities [17, 18]. Recent studies have shown that arctigenin can also suppress renal interstitial fibrosis, repress TGF-*β*-induced epithelial-mesenchymal transition (EMT) in human lung cancer cells, and attenuate PQ-induced EMT and pulmonary fibrosis [19–21]. Arctiin has also been reported to possess some similar biological functions [22–26]. However, the content of arctigenin in *Fructus Arctii* is much lower than that of arctiin. In our previous research, we found that arctiin could be metabolized into arctigenin in the intestinal tract [27, 28], and arctigenin was regarded to be the active form of arctiin. Based on these findings, we hypothesized that arctiin and arctigenin could ameliorate silica-induced oxidative injury in the lungs.

Herein, urine metabolomics was applied to explore the progress of silicosis with the aim at finding stable bioindices for the early diagnosis of silicosis and investigating the metabolic pathways in different phases of silicosis. Besides, the effects of arctiin and arctigenin on the progress of silicosis were compared to determine the more suitable compound for treatment. Network pharmacology and molecular docking were used to predict and analyze potential mechanisms for providing a theoretical basis for the development and utilization of arctiin and its metabolites.

## 2. Materials and Methods

### 2.1. Animals and Treatment

**Figure 1:**
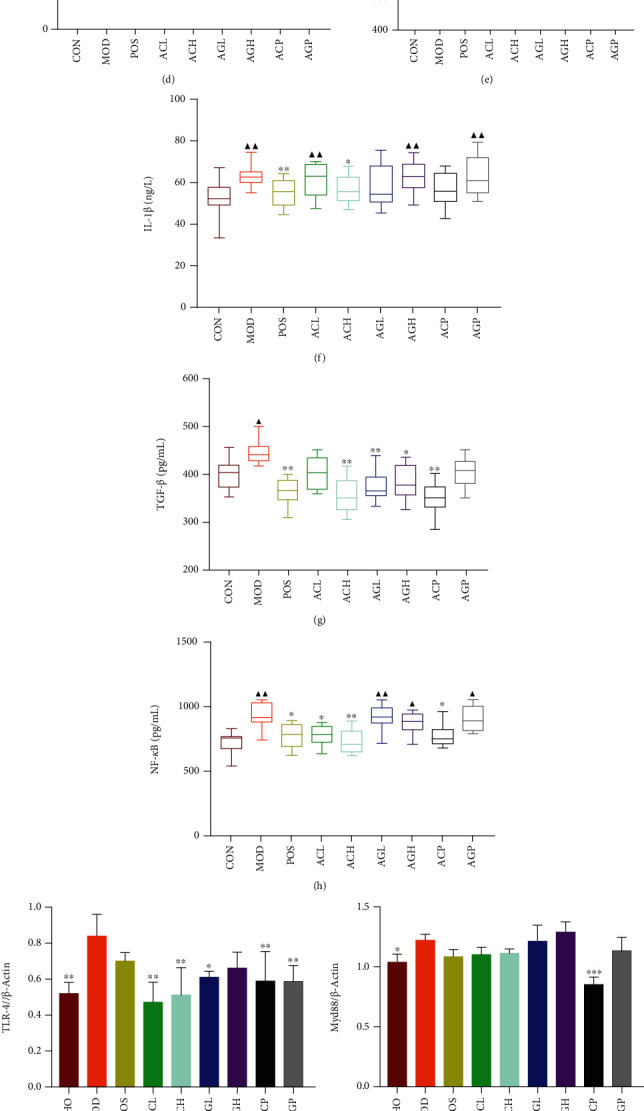
Experimental progress (a). The activities of ceruloplasmin (b) and lysozyme (c) in serum. The content of serum hydroxyproline (d). The level of TNF-*α*, IL-1*β*, TGF-*β*, and NF-*κ*B in lung tissues measured by ELISA (e–h). Note: mean ± SD, *n* = 8. ^▲^*P* < 0.05 and ^▲▲^*P* < 0.01 compared with CON. ^∗^*P* < 0.05 and ^∗∗^*P* < 0.01 compared with MOD. The expression of TLR-4, NLRP3, Myd88, ACS, NF-*κ*B, and *α*-SMA in lung tissues measured by Western blot (i–p). Note: mean ± SD, *n* = 3. ^∗^*P* < 0.05, ^∗∗^*P* < 0.01, and ^∗∗∗^*P* < 0.001 compared with MOD.

Adult male SPF level Wistar rats (weighing approximately 180–220 g) were obtained from the Liaoning Changsheng Biotechnology Co. Ltd., Liaoning, China (license key: SCXK (Liao) 20150001), housed in a specific pathogen-free environment, and maintained under controlled conditions (22 ± 2°C, 40 ± 10% relative humidity, and 12 h light/dark cycle) with free access to standard food and water. Animal research was approved by the Animal Ethical and Welfare Committee of Liaoning University of Traditional Chinese Medicine, and the experimental procedures were conducted according to the Guide for Care and Use of Laboratory Animals of Liaoning University of Traditional Chinese Medicine. The rats were modeled by tracheal intubation with 0.5 mL of 80 mg/mL SiO_2_ suspension (4000 U/mL penicillin sodium), and the model rats were evaluated via serum hydroxyproline and histopathological analysis. As described in Figure 1(a), after modeling, the rats in different groups were continuously intragastrically administrated with the corresponding drugs for 30 days. The rats were randomly divided into nine groups: the control group (CON, solvent), the model group (MOD, solvent), the tetrandrine-positive control group (POS, 30 mg/kg), the low-dose arctiin group (ACL, 30 mg/kg), the high-dose arctiin group (ACH, 60 mg/kg), the low-dose arctigenin group (AGL, 30 mg/kg), the high-dose arctigenin group (AGH, 60 mg/kg), the arctiin + positive group (ACP, arctiin 30 mg/kg and tetrandrine 30 mg/kg), and the arctigenin + positive group (AGP, arctigenin 30 mg/kg and tetrandrine 30 mg/kg). Further details of modeling and treatment are shown in Supplemental Materials.

### 2.2. Animal Behavior, Histological Assessment, and Mediator Measurements

Mental state, body weight, fur luster, respiratory rate, urine volume, dietary amount, and the amount of drinking water were monitored every week according to the methods specified in Supplemental Materials. Lung, spleen, and thymus tissues were collected and weighed to calculate their coefficient after drawing blood samples. Following this, the right part of the lung was used for the determination of bioindices and the left part was fixed in 4% paraformaldehyde solution, embedded in paraffin, and sectioned at a thickness of 5 *μ*m for histological evaluation via hematoxylin and eosin (H&E) and Masson's staining. The levels of hydroxyproline, ceruloplasmin, and lysozyme in the serum and several proinflammatory and profibrosis cytokines in lung tissues, including TNF-*α*, IL-1*β*, NF-*κ*B, and TGF-*β*, were determined by ELISA, and the expression levels of TLR-4, Myd88, NF-*κ*B p65, NLRP3, ASC, cleaved caspase-1, and *α*-SMA were assayed by Western blotting, aiming at evaluating the mechanism of arctiin and arctigenin against silicosis. Further details have been provided in Supplemental Materials.

### 2.3. Metabolomics Analysis

To explore the endogenous changes in the progression of silicosis, metabolomics was analyzed by HPLC-QTOF-MS (Agilent, USA), and data were preprocessed using MetaboAnalyst 4.0 (https://www.metaboanalyst.ca/).The normalized data were then subjected to Orthogonal Projections to Latent Structures Discriminant Analysis (OPLS-DA) in a Simca-p 14.1 workstation. Metabolites with VIP > 1 and *P* < 0.05 were identified as differential metabolites, and compound validation was conducted to verify the accuracy and eliminate unreliable differential metabolites. The metabolic pathways were identified by analyzing and enriching differential metabolites screened using the Pathway Analysis module in MetaboAnalyst 4.0. The MetScape plugin in Cytoscape 3.7.1 software was used for network construction of different periods and analysis of the topology of each network. Euclidean distance was used to evaluate the similarity between different periods by network topology and to determine the important metabolites in various periods [29]. A Venn diagram of metabolites in each period was displayed using the FunRich software, and changes in the same metabolite in different periods were analyzed. The diagnostic value was predicted using the ROC-based Biomarker Analysis module in MetaboAnalyst 4.0. Further details are presented in Supplemental Materials.

### 2.4. Effects of Arctiin and Arctigenin on Silica-Induced Macrophage Inflammation

To verify the effects of arctiin and arctigenin against inflammation, RAW264.7 murine macrophage cells were obtained from the Cell Bank of the Chinese Academy of Sciences (Shanghai, China). The cytotoxicity of arctiin and arctigenin toward RAW264.7 cells exposed to silica (50 *μ*g/mL) was assayed by MTT. The cells were then seeded in a 6-well plate at 1 × 10^6^ cells per well and incubated in DMEM containing 10% FBS for 24 h. The cells of the arctiin group (ARC) and the arctigenin group (ARG) were treated with 1 *μ*M arctiin or arctigenin and silica (50 *μ*g/mL) together for 24 h. The mitochondrial membrane potential and reactive oxygen species were detected using the assay kit, and the expression levels of iNOS, Arg-1, TLR-4, NLRP3, cleaved caspase-1, and TGF-*β* were evaluated. Further details are shown in Supplemental Materials.

### 2.5. Effects of Arctiin and Arctigenin on TGF-*β*1-Induced Myofibroblast Differentiation

To verify the effects of arctiin and arctigenin against fibrosis, primary mouse lung fibroblasts (PLFs) were isolated as described in Supplemental Materials. The cytotoxicity of arctiin and arctigenin on PLFs was assayed by MTT. The cells were seeded in a 6-well plate at 1.5 × 10^5^ cells per well and incubated in DMEM containing 10% FBS for 24 h. The cells were then treated with TGF-*β*1 (10 ng/mL) for 48 h. The cells were incubated with arctiin and arctigenin at 1 *μ*M. Following this, the expression level of *α*-SMA was measured by Western blotting. Additional details have been provided in Supplemental Materials.

### 2.6. Network Pharmacology-Predicted Pathway

Potential targets of arctiin and arctigenin were predicted by using TCMSP (http://lsp.nwu.edu.cn/tcmsp.php), STITCH (http://stitch.embl.de/), and SwissTargetPrediction server (http://www.swisstargetprediction.ch/), with stunted *Homo sapiens* as organism. CTD (https://ctdbase.org/) and GeneCards (https://www.genecards.org/) databases were used to find relevant target genes of pulmonary fibrosis. The compound-disease-target gene networks were constructed using the merge function of Cytoscape 3.7.1 software. Subsequently, the PPI network was established by STRING (https://string-db.org/) to select the closely connected genes for biological function analysis through FunRich software. The DAVID database (https://david.ncifcrf.gov/tools.jsp) was used to enrich the KEGG and GO pathway analyses of closely connected genes, and the enriched pathways were visualized on the OmicShare cloud platform (http://www.omicshare.com/forum/).

### 2.7. Molecular Docking Studies

For the preparation of the protein and the ligand, the crystal structure of TGF-*β*RI complexed with a 2-aminoimidazole inhibitor (PDB ID: 3faa) was downloaded from the Protein Data Bank (PDB), USA (https://www.rcsb.org/). Protein preparation was conducted using Discovery Studio 3.0 (BIOVIA, San Diego, California, USA); the ions, water molecules, and all internal ligands were removed; the missing atoms were inserted; and cocrystallization of small molecules was optimized before minimization of the target protein was conducted. The SDF documents of ligand compounds were downloaded from PubChem (https://pubchem.ncbi.nlm.nih.gov/), the initial molecular structure was optimized using Discovery Studio 3.0, and the molecular docking study was conducted using CDOCKER from Discovery Studio 3.0.

### 2.8. Prediction of Oral Bioavailability

AdmetSAR2.0 (http://lmmd.ecust.edu.cn/admetsar2/) was used to predict the oral bioavailability according to the Morgan fingerprint and random forest methods.

### 2.9. Statistical Analysis

One-way analysis of variance (ANOVA) was used for statistical analysis of the differences between groups. Data were analyzed statistically and have been expressed as mean ± standard deviation. IBM SPSS Statistics var. 17.0 (IBM Co., Armonk, NY, USA) was used to evaluate data. A value of *P* < 0.05 was considered statistically significant.

## 3. Results and Discussions

### 3.1. Arctiin and Arctigenin Attenuate Silicosis by Inhibiting TLR-4/NLRP3/TGF-*β* Signal Transduction

After rats were exposed to silica for 36 days, the surfaces of lung tissues were uneven locally, exhibiting a hard texture, poor elasticity, and increased volume, with a scattering of small white nodules (Figure Figure S1a). Through H&E staining, the lesions indicated inflammatory cell infiltration, pulmonary edema, edema in alveolar cavities, widened alveolar septa, increased pulmonary interstitial red blood cells, collagen deposition, and patchy fibrosis, showing a range of observations from mild inflammatory signs to fibrinous exudates and partial fibrous tissue proliferation. Masson's staining of MOD group lung sections showed a large blue area, indicating that the proliferation of collagen fibers was serious and that pulmonary fibrosis occurred (Figures S1b–S1e). Along with time, the appetite of rats in the MOD group became worse compared with the CON group, accompanied with low spirits, sluggish action, lusterless hair, irritability, tachypnea, and weight loss. This situation was in reversed tendency after treatment with arctiin and arctigenin (Figures S2a–S2f).

Serum lysozyme and ceruloplasmin were increased in dust-induced PF [30, 31] because lysosomes in cells were damaged and released lysozymes to stimulate fibroblast proliferation after macrophages swallowed the dust [32]. Ceruloplasmin promoted amino oxidation of lysine in peptide chains and eventually resulted in the formation of collagen fibrosis [33]. The level of hydroxyproline could indirectly reflect the degree of fibrosis because it was a unique amino acid in collagen [34, 35]. Compared with the CON group, the MOD group showed significantly increased levels of hydroxyproline, a specific marker of collagen fiber. The organ coefficient also reflected the reconstruction of lungs (Figures S2g–S2j). Serum ceruloplasmin and lysozyme were low in the CON group, but were increased significantly in the MOD group. After treatment, the activity of ceruloplasmin and the levels of hydroxyproline in the serum recovered to normal in different degree trends (Figures 1(b)–1(d)); particularly, the activity of ceruloplasmin was considerably restored in the AGP group.

Toll-like receptors (TLRs), a type of innate immunity pattern recognition receptor, can be triggered by endogenous danger signals released by macrophages, and the expression of TLR-4 can promote fibrosis formation [36, 37]. The increased expression level of Myd88, the main adaptor protein in the TLR-4 signaling pathway, could drive TAK1 via polyubiquitin chains generated by TRAF6 to activate NF-*κ*B [38, 39]. In contrast, the NF-*κ*B signaling pathway can mediate the synthesis of collagen; promote the transcription of cytokines such as TNF-*α*, IL-1*β*, and TGF-*β* [40]; and also induce chronic inflammation by activating the NLRP3 inflammasome [41–44]. The activation of inflammasome is essential for the inflammatory process leading to fibrosis [41]. Numerous inflammatory factors in chronic inflammation can regulate the fibrosis signal transduction cascade and induce fibrosis and malignant tumors [45–48]. However, TGF-*β* plays a central role in fibroblast activation and fibroblast differentiation into myofibroblasts [49–52], contributing to collagen production and extracellular matrix precipitation [53]. *α*-SMA is a myofibroblast-specific protein [54], while the synthesis of collagen is closely linked to hydroxyproline [55]. Compared with the MOD group, the CON group showed significantly low levels of immune cytokines TLR-4 and Myd88; proinflammatory cytokines TNF-*α*, IL-1*β*, NF-*κ*B, and NF-*κ*B p65; inflammasome proteins NLRP3, ASC, and cleaved caspase-1; profibrosis cytokine TGF-*β*; and fibrosis marker protein *α*-SMA in lung tissues. The increasing protein level in the MOD group indicated that the NLRP3 multiprotein was activated to stimulate the secretion of IL-1*β* via an immunoinflammatory response, and that the lung fibroblasts transformed into myofibroblasts after silica inhalation. The levels of TNF-*α*, IL-1*β*, NF-*κ*B, TGF-*β*, and *α*-SMA in the POS group; TNF-*α*, NF-*κ*B, TLR-4, NLRP3, and *α*-SMA in the ACL group; TNF-*α*, IL-1*β*, NF-*κ*B, TGF-*β*, TLR-4, NF-*κ*B p65, cleaved caspase-1, and *α*-SMA in the ACH group; TGF-*β* and TLR-4 in the AGL group; TGF-*β*, ASC, cleaved caspase-1, and *α*-SMA in the AGH group; TNF-*α*, NF-*κ*B, TGF-*β*, TLR-4, Myd88, NF-*κ*B p65, ASC, cleaved caspase-1, and *α*-SMA in the ACP group; and TLR-4 and ASC in the AGP group were significantly reduced compared with the levels in the MOD group (Figures 1(e)–1(p)).

### 3.2. Arctiin and Arctigenin Inhibit Oxidative Stress and Inflammation Induced by Silica in RAW 264.7 Cells

**Figure 2:**
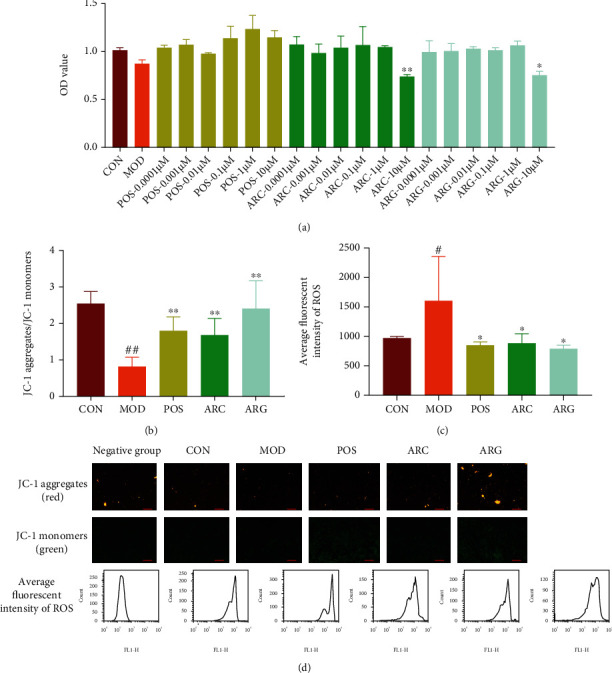
The toxicity of arctiin and arctigenin on macrophages (a). The mitochondrial membrane potential (JC-1 aggregates/JC-1 monomers) and the levels of ROS (b–d). Note: ^#^*P* < 0.05 and ^##^*P* < 0.01 compared with CON; ^∗^*P* < 0.05 and ^∗∗^*P* < 0.01 compared with MOD.

**Figure 3:**
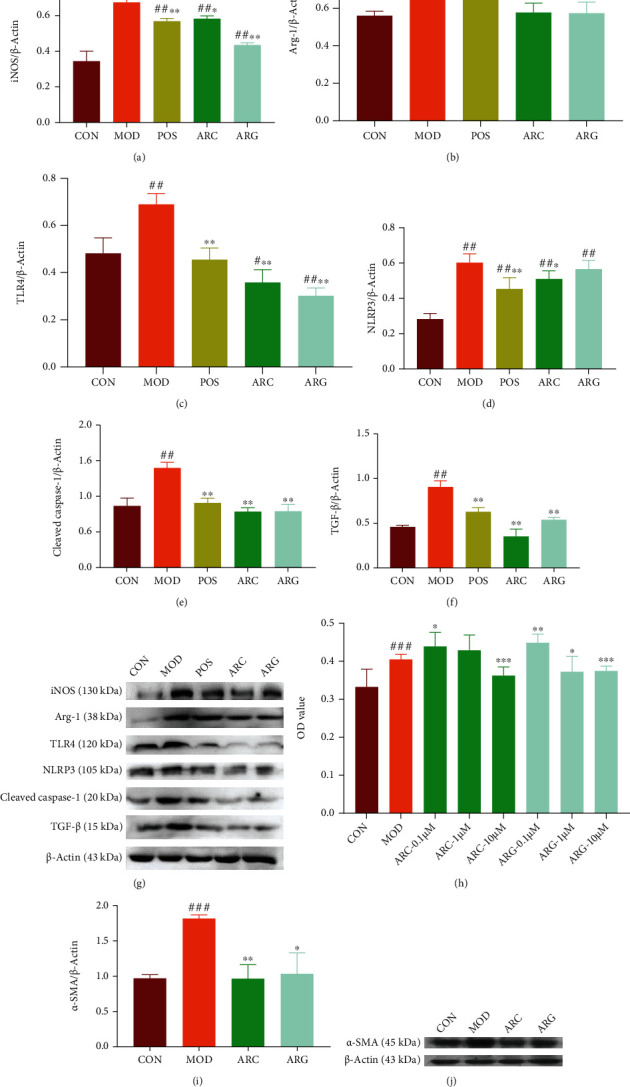
The expression of iNOS, Arg-1, TLR-4, NLRP3, cleaved caspase-1, and TGF-*β* by Western blot (a–g). The toxicity of arctiin and arctigenin on PFs (h). The expression of *α*-SMA by Western blot (i–j), mean ± SD, *n* = 3. Note: ^#^*P* < 0.05, ^##^*P* < 0.01, and ^###^*P* < 0.001 compared with CON; ^∗^*P* < 0.05, ^∗∗^*P* < 0.01, and ^∗∗∗^*P* < 0.001 compared with MOD.

Arctiin and arctigenin showed obvious cytotoxicity at 10 *μ*M and no cytotoxicity at 1 *μ*M in RAW 264.7 cells exposed to silica (Figure 2(a)). JC-1 aggregates/JC-1 monomers were decreased in the MOD group compared with the CON group, whereas the ARG group was better than the ARC group for their increase (Figures 2(b) and 2(c)). Levels of ROS displayed a contrary tendency, rising in the MOD group but declining much more in the ARG group than in the ARC group (Figures 2(b) and 2(d)). We concluded that silica could induce macrophage polarization and could increase the expression levels of iNOS and Arg-1, representing that the M0 of macrophages polarized to M1 and M2. Besides, the levels of TLR-4, NLRP3, cleaved caspase-1, and TGF-*β*1 were increased, indicating that cell inflammation occurred on macrophage polarization. Our results indicated that the increased expression levels of iNOS and Arg-1 induced by silica in the ARC group and the ARG group were both downregulated, indicating that the effects of the ARC group and the ARG group on silicosis may be associated with the blocking of the TLR-4/NLRP3/TGF-*β* signaling pathway (Figures 3(a)–3(g)).

### 3.3. Arctiin and Arctigenin Inhibit TGF-*β*1-Induced Myofibroblast Differentiation

Arctiin showed no obvious cytotoxicity on PLFs at 1 *μ*M, whereas arctigenin was cytotoxic (Figure 3(h)). Both the ARC group and the ARG group could reduce the expression of *α*-SMA, which was increased by myofibroblast differentiation induced by TGF-*β*1. However, arctiin had a better effect than arctigenin given its lower toxicity (Figures 3(i) and 3(j)).

### 3.4. Silicosis Progression Based on Metabolomics

Day 7, day 21, and day 35 were defined as the early, middle, and final phases of silicosis. Differences between the MOD group and the CON group increased gradually with the progress of silicosis on OPLS-DA (Figure S3a). In all, 56 metabolites in the early phase, 58 metabolites in the middle phase, and 52 metabolites in the final phase were identified by targeted and untargeted metabolomics. Further, 27, 25, and 33 of these exhibited significant differences (Tables S1–S4).

**Figure 4:**
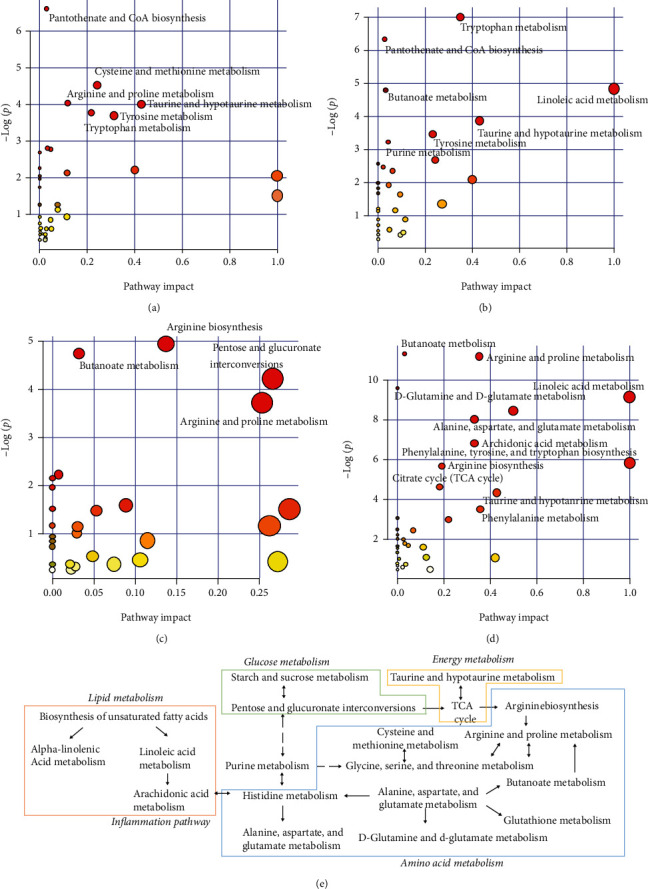
Metabolic pathway analysis by MetaboAnalyst: day 7 urine (a), day 21 urine (b), day 35 urine (c), day 36 serum (d), and metabolic pathway network (e).

Metabolic pathways indicated that the early phase of silicosis was associated with pantothenate and CoA biosynthesis, cysteine and methionine metabolism, and taurine and hypotaurine metabolism. The middle phase was closely associated with tryptophan metabolism, pantothenate and CoA biosynthesis, and linoleic acid metabolism among others. The final phase was associated with arginine biosynthesis, butanoate metabolism, and arginine and proline metabolism (Figures 4(a)–4(d)). The metabolic pathway network was established according to the KEGG database, indicating that the mechanism underlying the development of silicosis was closely associated with inflammation, oxidative stress, and lipid and amino acid synthesis and decomposition (Figure 4(e)).

According to the pathway analysis of urine metabolites in various phases of silicosis, we found that in the early and middle phases of silicosis, leukotriene B4 and L-cysteine, which might be associated with inflammation and antioxidant mechanisms, were upregulated. The self-protection process of the organism was initiated, and the antioxidant and immune functions were improved to antagonize external stimulation [56]. In the middle phase, upregulated PC and LysoPC may be associated with the destruction of cell membrane structure by silica, causing lipid peroxidation [57]. Arginine and proline metabolism, which promoted hydroxyproline synthesis, was activated, reflecting the presence of collagen synthesis and the occurrence of fibrosis [58, 59].

Based on the abovementioned research results of metabolomics and signal transduction, we found that oxidative stress, inflammation, and enhanced innate immune responses were the main reactions after silica was phagocytosed by macrophages in the early phase. NLRP3 inflammasome was activated through the TLR-4/Myd88/NF-*κ*B cascade, promoting the secretion of inflammatory factors IL-1*β* and TNF-*α* and the fibrogenic factor TGF-*β*. Substance metabolism was guided and regulated through signal transduction, and signal molecules activated a series of cascade reactions by binding with receptors to transmit external information to intracellular regions, affecting substance metabolism, gene expression, or biological functioning of cells. Metabolomics indicated that arginine and proline metabolism made a considerable contribution to the synthesis of collagen. Therefore, gene PPI interaction analysis of arginine and proline metabolism and TLR-4/NLRP3/TGF-*β* signal transduction was investigated to elucidate the relationship between substance metabolism and signal transduction. Figure S4 shows that there are six related genes between TLR-4/NLRP3/TGF-*β* signal transduction and arginine and proline metabolism, including ARG1, ARG2, NOS1, NOS2, NOS3, and ALDH7A1. NOS2 (iNOS) and Arg-1 were macrophage polarization markers of M1 and M2 macrophages, respectively. The NLRP3 inflammasome could mediate M1 macrophage polarization to promote the production of IL-1*β* and upregulate the expression of IL-4 to stimulate M2 macrophage polarization [60–62]. Arctiin and arctigenin could both inhibit macrophage M0 polarization to M1, alleviate proinflammatory response, and reduce the release of inflammatory factors. The polarization of M1 to M2 was also blocked; this may be because of a decrease in the level of M1 macrophages. The blockage of macrophage polarization may be associated with the blocking of TLR-4/NLRP3/TGF-*β* signaling.

### 3.5. Topological Analysis of Metabolic Network in Different Phases and the Prediction of Diagnostic Indices of Silicosis

**Figure 5:**
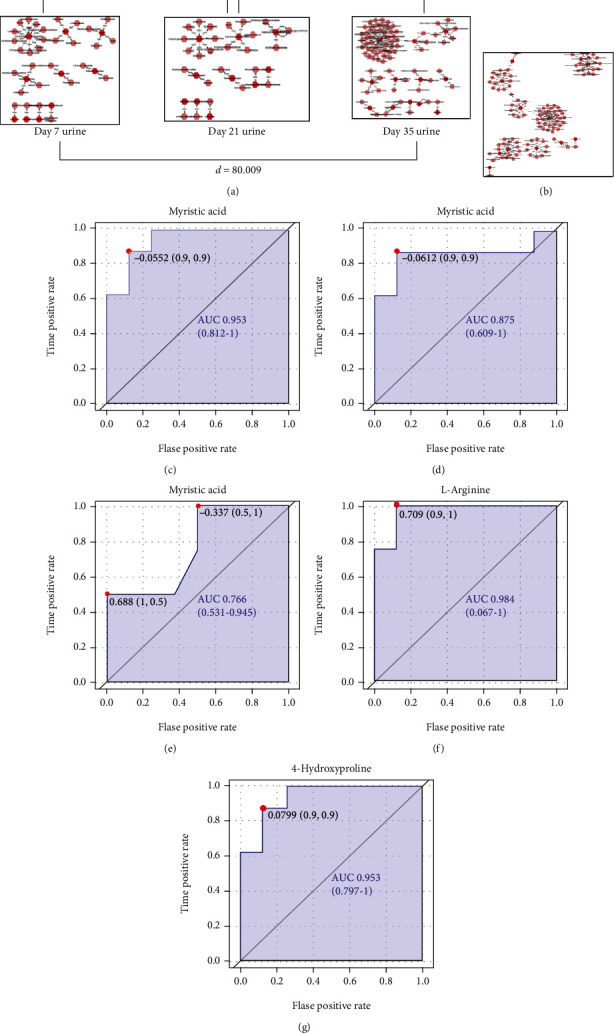
Metabolic network and the prediction of diagnostic indexes. Topological analyses of urine metabolic network (a) and serum metabolic network (b). ROC analysis of myristic acid, L-arginine, and 4-hydroxyproline (c–g).

The metabolic networks in different periods of silicosis are displayed in Figures 5(a) and 5(b). Urine metabolic network topology parameters (Table S5) were used to calculate the *d* value, representing dissimilarity, according to the Euclidean Distance Formula. This means that the smaller the *d* values are, the more similar the networks are.  Figure 5(a) shows that the similarity between the early and final phases was the largest; the similarity between the early and middle phases was smaller than that between the early and final phases. After the overall evaluation of different phases, the degree and betweenness centrality were used to further investigate the importance of nodes in different phases. In this study, myristic acid in the urine was selected as a critical metabolite, L-arginine and 4-hydroxyproline in serum were also prominent in the network (Table S6).

11 metabolites were coexisting in urine during different periods in the MOD group compared with the CON group. The levels of myristic acid, uracil, mevalonic acid, 3-hydroxybutyric acid, pantothenic acid, epinephrine, and succinic acid semialdehyde were downregulated, whereas those of 3,6-octadienoylglycine, 1-methyluric acid, 4-hydroxyproline, and 1-methylhistidine were upregulated in a time-dependent manner (Table 1). Myristic acid, 3,6-octadienoylglycine, and uracil in the early phase; myristic acid, 3,6-octadienoylglycine, 3-hydroxybutyric acid, and pantothenic acid in the middle phase; and myristic acid, 3,6-octadienoylglycine, 3-hydroxybutyric acid, and 1-methyluric acid in the final phase satisfied the differential metabolite requirements for VIP > 1 and *P* < 0.05 in metabolomics. Subsequently, ROC analysis was conducted, and the diagnostic value of metabolites was evaluated with the AUC cut-off of 0.9. The AUC of myristic acid was up to 0.953, indicating a high diagnosis value in the early phase, and it reduced to 0.875 and even 0.766 gradually with the development of silicosis (Figures 5(c)–5(e)). Besides, the AUCs of serum L-arginine and 4-hydroxyproline were also high, at 0.984 and 0.953 respectively (Figures 5(f) and 5(g)), and they may be effectively used as biomarkers for pulmonary fibrosis.

Myristic acid is a type of saturated fatty acid [63]. Proteins can acylate through the N-terminal myristoylation reaction of myristic acid, which has a membrane localization effect. This membrane localization effect can promote NO release from the cells, and the oxidative stress of NO can help macrophages against the pathogens in the immune system. However, unmyristoylated proteins bind to the receptor CD36 and then activate NOS by influencing AMP kinase through Src kinase [64]. The importance of NOS in arginine and proline metabolism also reflects the indirect contribution of myristic acid to arginine and proline metabolism. Further, the content of hydroxyproline, which is a unique amino acid in collagen fibrosis, can reflect the degree of fibrosis [65]. The level of urine hydroxyproline in the early phase of silicosis showed no difference when compared with the level in the CON group, representing that there was no fibrosis during that time, whereas upregulating trends were observed in the middle and final phases of silicosis. Particularly, significantly different levels were observed in the serum, representing fibrosis induced by the formation of silica. Initially, fibrosis was evaluated by hydroxyproline alone. This study further revealed that the dynamic changes in myristic acid or 4-hydroxyproline could reflect the degree of fibrosis: myristic acid was downregulated and 4-hydroxyproline was upregulated with the progression of silicosis. However, the effect of arctigenin on myristic acid was not as good as that of arctiin in the final phase of silicosis, mostly because of the toxicity of arctigenin as a result of *in vivo* accumulation with long-term administration [66, 67]. Results of the MTT assay of cells also reflected that the toxicity of arctigenin was higher than that of arctiin at the same concentration. Besides, neither arctiin nor arctigenin showed significant regulatory effects on L-arginine, but both arctiin and arctigenin could downregulate 4-hydroxyproline compared with the levels in the MOD group. L-Arginine and 4-hydroxyproline both participated in arginine and proline metabolic pathways, but L-arginine was not involved in the synthesis of collagen.

### 3.6. Effects of Arctiin and Arctigenin on the Different Phases of Silicosis by Metabolomics

**Figure 6:**
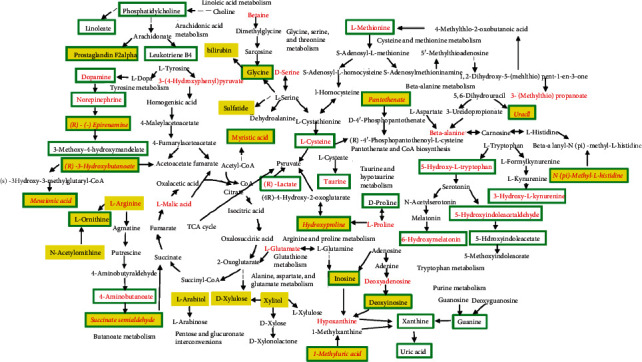
Urine metabolic network (note: early metabolites in red, middle metabolites in yellow, and final metabolites in green).

The metabolic networks are displayed in Figure 6. OPLS-DA results displayed as score plots could indicate the scatter of the samples, wherein similar metabolomic compositions were clustered together and compositionally different metabolomic compositions were dispersed. The score plots of urine showed that the ACL group and the ACH group were more similar to the CON group, whereas the AGL group and the AGH group were closer to the MOD group, indicating that the ACL group and the ACH group could regulate metabolic disorders effectively. The OPLS-DA results of serum metabolism showed a similar trend (Figure S3b).

Based on the abovementioned results, myristic acid in urine had a higher diagnostic value in the early phase, and L-arginine and 4-hydroxyproline in the serum had higher diagnostic values in the final phase of silicosis. Therefore, the regulatory effects of arctiin and arctigenin on these high-value metabolites could evaluate their efficacy. The results showed that arctiin and arctigenin could both modify myristic acid levels in the early phase with similar strength, and arctigenin had a better effect on myristic acid in the middle phase than arctiin, whereas arctiin had a better effect than arctigenin in the final phase of silicosis. Further, the efficacy of a combined medication with arctiin and positive drugs was better than that of arctiin alone, showing a synergistic effect. There was no obvious regulatory effect of arctiin and arctigenin on L-arginine in the serum, but the efficacy was increased with combined medication using arctiin and positive drugs. Arctiin had a better effect on serum 4-hydroxyproline than arctigenin (Tables S1–S4).

Compared with the MOD group, the arctiin groups showed significant differences in 11 of 56 urine metabolites in the early phase, 8 of 58 urine metabolites in the middle phase, and 15 of 52 urine metabolites and 16 of 46 serum metabolites in the final phase. In contrast, there were 16 of 56 urine metabolites in the early phase, 11 of 58 urine metabolites in the middle phase, and 22 of 52 urine metabolites and 24 of 46 serum metabolites in the final phase that showed significant differences after treatment with arctigenin. It can be clearly concluded that arctigenin regulated more metabolites than arctiin. The pathways of these abovementioned metabolites indicated that arctiin could regulate the biosynthesis of hormones in the early phase; taurine and hypotaurine metabolism in the middle phase; and taurine and hypotaurine metabolism, arginine and proline metabolism, glycerophospholipid metabolism, and phenylalanine, tyrosine, and tryptophan biosynthesis in the final phase of silicosis, whereas arctigenin could regulate arginine and proline metabolism and synthesis and degradation of ketone bodies in the early phase; linoleic acid metabolism in the middle phase; and linoleic acid metabolism, pantothenate and CoA biosynthesis, *β*-alanine metabolism, arachidonic acid metabolism, butanoate metabolism, arginine and proline metabolism, alanine, aspartic acid and glutamic acid metabolism, and the metabolism of glycine, serine, and threonine in the final phase of silicosis (Tables S1–S4). Above all, arctiin and arctigenin could regulate energy metabolism and glucose metabolism by intervening in lipid metabolism and inflammation-related pathways and inhibit collagen synthesis to attenuate silicosis.

### 3.7. Network Pharmacological Analysis to Determine the Regulatory Effect of Arctiin and Its Metabolites on Silicosis

The network pharmacology of arctiin and its 15 metabolites was performed, and pathway enrichment results of network pharmacology showed the TNF signaling pathway, the NOD-like receptor signaling pathway, Toll-like receptor signaling, the MAPK signaling pathway, and 90 related pathways, indicating that arctiin and its metabolites play important roles in the treatment of silicosis. Biological function analysis further indicated that the regulatory effect of arctiin and its metabolites on silicosis was associated with biological processes such as the lipopolysaccharide-mediated signaling pathway; positive regulation of the ERK1 and ERK2 cascade; cellular response to mechanical stimulus; and molecular functions such as interleukin-1 receptor binding, MAP kinase activity, and ATP binding (Figure S5), providing a new direction for the development of drugs. Research indicated that ATP, which might be relied on energy metabolism or glucose metabolism, was needed for the activation of the NLRP3 inflammasome [68].

### 3.8. Oral Bioavailability Prediction and Molecular Docking Studies of Arctigenin and Its Metabolites

Arctiin and arctigenin could inhibit the conversion of fibroblasts to myofibroblasts and reduce the accumulation of extracellular matrix *in vitro*; however, arctiin was better than arctigenin for the treatment of silicosis being associated with the toxicity of arctigenin. Arctigenin was mostly regarded as the direct effective compound of arctiin in *Fructus Arctii*, but its oral bioavailability is lower because of its lignan structure and insolubility in water, and it is usually administered in the form of an injection [69]. Arctiin is present in a much higher concentration than arctigenin in *Fructus Arctii* [70]. Our results indicate that arctiin can be hydrolyzed into arctigenin and has various pharmacological effects given its high polarity and easy oral absorption [71, 72]. The prediction of oral bioavailability of arctiin was 75%, whereas that of arctigenin was 58% according to AdmetSAR2.0 web-service (Table S7), indicating that arctiin is easier as a medicine because of its high oral bioavailability. Besides, arctiin and its metabolite 4′,4^″^-dihydroxyl-enterolactone showed better binding with TGF-*β*RI according to the XP Gscore in molecular docking studies (Table S8). A previous study on the pharmacokinetics of arctiin after oral administration indicated that it had a rapid absorption phase followed by a sharp but lasting disappearance, which indicates that it could be metabolized into arctigenin and other metabolites [28]. Thus, arctiin coexists with lower concentrations of its metabolites being produced by metabolizing arctiin constantly in the organism after oral administration. Compared with the toxicity of arctigenin alone on oral administration, that of arctiin was lower because it constantly produced low concentrations of arctigenin. Thus, arctiin showed a much higher druggability than arctigenin because of its high oral bioavailability and low toxicity.

## 4. Conclusions

To the best of our knowledge, this is the first study identifying urine myristic acid and serum L-arginine and 4-hydroxyproline as biomarkers for silicosis diagnosis. The mechanism underlying the development of silicosis is closely associated with inflammation, oxidative stress, and lipid and amino acid synthesis and degradation. Arctiin and arctigenin could ameliorate oxidative stress, immune-related inflammatory reaction, and fibrosis through pantothenate and CoA biosynthesis, cysteine and methionine metabolism, taurine and hypotaurine metabolism, linoleic acid metabolism, and arginine and proline metabolism successively. The importance of macrophage polarization in fibrosis was verified by integrated arginine and proline metabolism and the TLR-4/NLRP3/TGF-*β* signaling pathway, and arctiin and arctigenin could both attenuate the development of silicosis and protect the lungs from injury by blocking the polarization of macrophages and inhibiting the differentiation of myofibroblasts by regulating TLR-4/NLRP3/TGF-*β* signal transduction. Our research for the first time reveals that both arctiin and arctigenin are effective in silicosis, with the former showing better druggability.

## Figures and Tables

**Table 1:** The mutual metabolites in urine of various periods.

Metabolites	Day 7		Day 21		Day 35	
	Trend	VIP	Trend	VIP	Trend	VIP
Myristic acid	+++	3.461	++	1.509	++	2.133
3,6-Octadienoylglycine	++	2.105	--	1.946	++	1.828
Uracil	++	1.968	/	0.063	+	1.161
Mevalonic acid	+	1.663	/	0.021	/	0.189
3-Hydroxybutyric acid	-	1.078	--	1.501	--	1.943
Pantothenic acid	+	0.824	++	1.409	/	0.528
1-Methyluric acid	-	0.822	/	0.092	++	1.723
Epinephrine	+	0.578	+	0.924	/	0.055
4-Hydroxyproline	/	0.299	+	1.119	+	1.106
Succinic acid semialdehyde	/	0.252	-	0.241	-	0.327
1-Methylhistidine	-	0.213	/	0.004	+	1.148

Note: “-” represents metabolites with a downregulated trend, “+” represents metabolites with an upregulated trend, “--” and “++” represent significant difference from control at *P* < 0.05, and “---” and “+++” represent significant difference from control at *P* < 0.01.

## Data Availability

The metabolomics data and molecular docking data used to support the findings of this study are included within the supplementary information file, and other data used to support the findings of this study are available from the corresponding author upon request.

## Supplementary Materials

Supplementary Materials More details and procedures including materials, dose selection, modeling methods, metabolomics, and cell cultures and some figures and tables to support the results and conclusion are shown in Supplemental materials.
