# Supplementary material for: The Ameliorative Effects of Arctiin and Arctigenin on the Oxidative Injury of Lung Induced by Silica via TLR-4/NLRP3/TGF-β Signaling Pathway
Source: Oxid Med Cell Longev. 2021 Jul 17;2021:5598980. doi: 10.1155/2021/5598980 (PMC8313330; doi:10.1155/2021/5598980)
Supplement: Supplementary Materials — More details and procedures including materials, dose selection, modeling methods, metabolomics, and cell cultures and some figures and tables to support the results and conclusion are shown in Supplemental materials. [file 5598980.f1.pdf]

Supplemetal materials and Methods

Materials

Arctiin and arctigenin were separated and purified by our laboratory and the final product shown an over 98% purity by HPLC detection. Trandrine tablets were provided by Zhejiang Jinhua Conba Bio-Pharm. CO., LTD. China. Silica (SiO_2 purity >99%, particle size 0.5-10 μm (approx. 80% between 1-5 μm) was purchased from Sigma, St., Louse, MO, USA. Penicillin G was suppied by North China Pharmaceutical Co., Ltd. 2-ketoglutaric acid, pyruvic acid, succinic acid, disodium fumarate, malic acid, stearic acid, 3-hydroxytyramine hydrochloride and mixed amino acid standards were purchased from Sigma, USA. Betaine, allantoin, inosine, taurine, creatinine, L-carnitine, creatine, urea and citric acid were recruited from Aladdin, USA. Sodium lactate, doxifluridine, D-(+)-pantothenic acid calcium salt and 6-hydroxypurine were purchased from the National Institutes for Food and Drug Control, China. TNF-α, IL-1β, NF-κB and TGF-β ELISA kit (FANKEL BIO, Shanghai, China), Hydroxyproline (HYP), ceruloplasmin and lysozyme assay kits (Jiancheng Bioengineering Institute, Nanjing, China). The mitochondrial membrane potential assay kit (Beyotime Biotechnology, Shanghai, China.), reactive oxygen species assay kit (Jiancheng Bioengineering Institute, Nanjing, China.), SDS-PAGE Sample Loading Buffer (Beyotime, Shanghai, China), BCA Protein Assay Kit (Beyotime, Shanghai, China). Antibodies against the following proteins were used: α-SMA, NF-κB, TLR-4, Myd88, NLRP3, ASC, cleaved Caspase-1 (Wanleibio, Shenyang, China), β-actin (Absci, USA), HRP-conjugated goat antirabbit IgG (Wanleibio, Shenyang, China), BeyoECL Plus (Beyotime, Shanghai, China).

Dose selection

The daily dose interval of *Fructus Arctii* is 6 - 12 g according to Chinese Pharmacopoeia, in which the arctigenin account for 5% of *Fructus Arctii*. In this study, we selected the human dosage of 6 g/day (equivalent dosage of 300 mg/kg/day of arctigenin and about 30 mg/kg/day for rats equivalent dosage of arctigenin) as the low-dose reference and 60 mg/kg/day for the high-dose reference to investigate the therapeutic effect of different doses of arctigenin in silicosis rats.

Tetrandrine is widely used for silicosis therapy in clinical in China. The instruction of tetrandrine tablets indicated the dose against silicosis is 60mg-100mg/times, 3 times everyday. In this study, we selected the human dosage of 300mg daily (equivalent dosage of 31.25mg/kg/day for rats) and decided 30mg/kg/day as the positive drugs dosage.

Modeling methods

Before modeling, rats were randomly equally divided into 9 groups, i.e. the control group (CON), the model group (MOD), positive control group (POS), low dose group of arctiin (ACL), high dose group of arctiin (ACH), low dose group of arctigenin (AGL), high dose group of arctigenin (AGH), arctiin + positive group (ACP) and arctigenin + positive group (AGP). Rats were modeled by tracheal intubation as the methods as follows. Rats were anesthetized by injecting 3% pentobarbital sodium (1 mL/kg body weight) in the abdominal cavity, then fixed limbs and placed on a board facing upward at an angle of approximately 45° by using elastic string carefully positioned under the animal's front incisors. The tongue was gently pulled out with forceps and sterilized stomach tube (2.0 mm diameters) was intubated into the trachea. Modeling suspension was instilled into the trachea via the catheter, then air injection to make sure suspension completely access into the trachea. Then whirling rats to make the drug distributed in vivo uniformly. The sham-operation group was given sterile saline with penicillin in the same operation. After modeling, the rats in different groups were continuously intragastrically administrated with the corresponding drug for 30 days.

Mental state evaluation standard: lively-0, slightly slow response and voluntary activity reduced-1, laziness, mental atrophy, curled up limbs, slow movement-2, malaise, aggressive and confrontational behavior disappeared-3. The respiratory rate was evaluated through the animal breaths in 1 min under quiet and repeat 3 times. The amount of dietary and drinking water was caculated by weight lost method.

Urine and serum samples preparation

Urine samples were collected by metabolism cages at first week, thrid week and fifth week and the volume were recorded. All groups were weighted and then anesthetized

at 24 hours after the last administration, blood samples were drawn from every animal abdominal aorta and the serum was harvested by centrifuge. Serum and urine samples were prepared respectively according to the protocol as follows. Urine was centrifugated to remove particulates, then diluting with water (1:1) (brief vortex) vol/vol and stored at 4 °C before analysis. Serum was diluted with acetonitrile (1:2.5) (brief vortex) vol/vol and centrifugated to remove particulates. Then all the supernatant was removed and evaporated to dryness under nitrogen. The dry residue was reconstituted in acetonitrile and water mixture (1:1) (brief vortex), then centrifuged again at 100g for 3 min at 4 °C. All supernatant was collected and transferred into new vials and ready for UPLC/TOFMS analysis.

Chromatography conditions

Metabolic profiling Chromatography: Chromatography was performed using a HPLC system (Agilent1290, USA) equipped with a TOF mass spectrometer (G1969A, Agilent). An aliquot of 2 μL of the serum sample solution (urine sample 5μL) was injected onto an ACQUITY UPLCTM HSS T3 column (100 mm×2.1 mm, 1.7 μm Waters Corp, Milford, USA) at 40°C with a flow rate of 0.40 mL/min (urine sample 0.50 mL/min). The mobile phases were composed of 0.1% formic acid in water (solvent A) and 0.1% formic acid in acetonitrile (solvent B), the gradient was used as follows: Next an aliquot of 2 μL of the serum sample solution was injected onto an Poroshell 120 HILIC-Z column (100 mm×2.1 mm, 2.7 μm, Agilent Technologies, USA) at 40°C with a flow rate of 0.40 mL/min. The mobile phases were composed of 10% ammonium acetate in water (solvent A) and, 10% ammonium acetate in acetonitrile (solvent B), the gradient was used as follows: Gradient elution program. The eluant was introduced to the mass spectrometer directly and analyzed with positive/negative electrospray ion source (ESI). The quality control (QC) sample was used to optimize the condition of UPLC-MS, as it contained most information on whole samples. After every 9 samples injected, a pooled sample as the QC sample followed by a blank was injected in order to secure the stability and repeatability of the UPLC-MS systems.

Mass spectrometry conditions: Source gas temperature was 350 °C. Fragmentor voltage

was 380 V. OCT IRF Vpp was 750 V. Vcap pressure was 4000 V (ESI+)/3500 V (ESI-). Nebulizer pressure was 35 psi. Drying gas flow was 15 L/min. Drying gas temperature was 200℃. The data acquisition rate was set to 5.0 Spectra·s^{-1} and the mass range was set at *m/z* 50 -1200 using extended dynamic range.

Data processing and analysis

The MassHunter Workstation software (version B.04.01 Qualitative nalysis, Agilent) was used for feature extraction by the Molecular Feature Extractor (MFE) algorithm. The extracted compound list of QC was exported as Compound Exchange Format (.cef) file as target template for data extraction by Profinder (Version B. 08. 00, Agilent). The extracted data were export as EXCEL (.csv) file for further statistical analysis. In Simca-p 14.1 Workstations, the normalized data were then subjected to OPLS-DA. As a supervised pattern recognition method, PLS-DA could reflect the difference of distinct groups. It is a valuable visualization tool which we can show the different groups separated in the three-dimensional space.

Module difference analysis

In this study, the MetScape plugin in Cytoscape software was used for network construction (parameters: build pathway-based network; organism: human; networktype: compound). Cytoscape and Network Analyzer were used to visualize the network and analyze the topology of this network. Six dimensions of topology parameters, including network centralization、Avg. number of neighbors、characteristic path length、 network density、 numbers of nodes and numbers of edges were selected to represent the network, and Euclidean distance was used to integrate these different dimensions.That is, assuming a is the structure of network A, whose topological state was described by its characteristics of space vector using the six dimensions of the topology parameters, namely, a1, a2, a3... a6. After the drug intervention, the topology of network A may change to network B, and the corresponding topological structure b was b1, b2, b3... b6. Notably, we defined the overlap structure (the same nodes and edges) of network A and B as the original nodes and edges, named (a7, a8) and (b7, b8). Here, the value of (a7, a8) is actually (0, 0) and (b7, b8) is the nodes and edges of network B from network A. The formula is as follows:

$$d = \sqrt{(a_1 - b_1)^2 + \cdots (a_8 - b_8)^2}$$

Western blotting analysis

The preparetion of protein samples: The lung tissues and cells homogenates were prepared respectively by using RIPA Lysis Buffer containing protease and phosphatase inhibitors. Total protein content was determined by the BCA Protein Assay Kit according to manufacturer's instructions. Samples were stored at -80°C until analysis. Western blot protocol: Before loaded, samples were denatured in SDS-PAGE Sample Loading Buffer by boiling for 5 min. Equal amount of protein extracts (25 mg) were subjected to 6-12% sodium dodecyl sulfate-polyacrylamide gel electrophoresis (SDS PAGE) and transferred to PVDF membrane. The PVDF membranes were then blocked in 5% nonfat milk for 2 hours at room temperature. The membranes were incubated with the specific primary antibodies. After washing the membranes with 1×TBST for 3 times, the slices were incubated with HRP-conjugated goat antirabbit IgG (1:7000 diluted with 1×TBST) for 2 hours at room temperature. We used ECL reagents to detect immunoreactive bands.

Isolation and culture of primary mouse lung fibroblasts

The primary mouse lung fibroblasts (PLFs) were isolated from as described previously, with some modifications [22]. Briefly, Lung tissue collected from C57/BL6 mice (Liaoning Changsheng Biotechnology Co. Ltd, China. License Key: SCXK (Liao) 20150001) were minced into 1 to 2 mm^3 pieces and digested with trypsin for 15 min at 37 °C. The digested cell suspensions were collected and cultured with DMEM complete medium and incubated at 37 °C in an atmosphere of 5% CO_2 in air over night. After cells attached, the medium was changed the next day. The percentage of fibroblast was identified as 95% by morphology under microscope.

The cell cytotoxicity assay of arctiin and arctigenin

Cell cytotoxicity was assayed by the MTT assay. Briefly, RAW264.7 were seeded in 96 well at 1×10^5 per well and incubated in DMEM containing 10% FBS for 24 h. Then were treated with SiO_2 (50 μg/mL) and some of them were incubated with arctiin and arctigenin at different concentrations (10, 1, 0.1, 0.01, 0.001 μM) for 24 h at 37 °C in

an atmosphere of 5% CO_2 in air. Then 5 mg/mL MTT was added to the wells and incubated for additional 4 h at 37 °C in an atmosphere of 5% CO_2 in air. After mixwell, the optical density was measured at 490 nm. And PLFs were seeded in 96 well at 5×10^4 cells per well and incubated in DMEM containing 10% FBS for 24 h. Then were treated with TGF-β1 (10 ng/mL) for 48 h and some of them were incubated with arctiin and arctigenin at different concentrations (100, 10, 1, 0.1 μM). Then MTT detection was consistent with above.

Mitochondrial membrane potential detection

RAW264.7 cells incubated with arctiin or arctigenin at 1μM and silica (50 μg/mL) together for 24 h coated 6-wells were collected and resuspended in fresh medium. After the addition of 1 mL JC-1 working solution, the cells were incubated at 37 °C in an atmosphere of 5% CO_2 in air for 20 min. The staining solution was removed by centrifugation and cells were washed with JC-1 staining buffer twice, then resuspended in fresh medium. Mitochondrial membrane potentials were monitored by determining the relative amounts of dual emissions from mitochondrial JC-1 monomers or aggregates using an Motic fluorescent microscope.

Reactive oxygen species detection

RAW264.7 cells incubated with arctiin or arctigenin at 1μM and silica (50 μg/mL) together for 24 h coated 6-wells were collected and resuspended in DCFH-DA probes diluted in PBS. Then the cells were incubated light-avoided at 37 °C in an atmosphere of 5% CO_2 in air for 60 min and agitated every 5 min to make cells fully contact with probes. After that, cells were obtained through centrifugation and washed 3 times with PBS, then resuspended in 500μL PBS. The production of ROS was determinated through flow cytometry (Excitation wavelength 485 nm, emission wavelength 525 nm).

Supplemental Figure Legends

Figure S1 Lung tissues (a), H&E (b. 10×10, c. 20×10) and Masson (d. 10×10, e. 20×10) staining observation.

Figure S2 Mental state, body weight, fur luster, respiratory rate, urine volume, dietary amount and the amount of drinking water of rats (a-f). Organ coefficient (h. Lung

coefficient, i. spleen coefficient, j. thymus coefficient) Note：mean ± SD, n = 8, ▲*P* <0.05, ▲▲*P* <0.01 *vs* CON；**P* <0.05, ***P* <0.01 *vs* MOD.

Figure S3 OPLS-DA plot of metabolomics (a. CON and MOD, b. All groups).

Figure S4 Arctiin and arctiin metabolites - target - PF interaction (a)，PPI network (b)，GO biological process (c), GO molecular function (d), KEGG pathway (e), Reactome pathway (f).

Figure S5 PPI network of related genes of argrine and proline metabolism and TLR-4 / NLRP3 /TGF-β signaling.

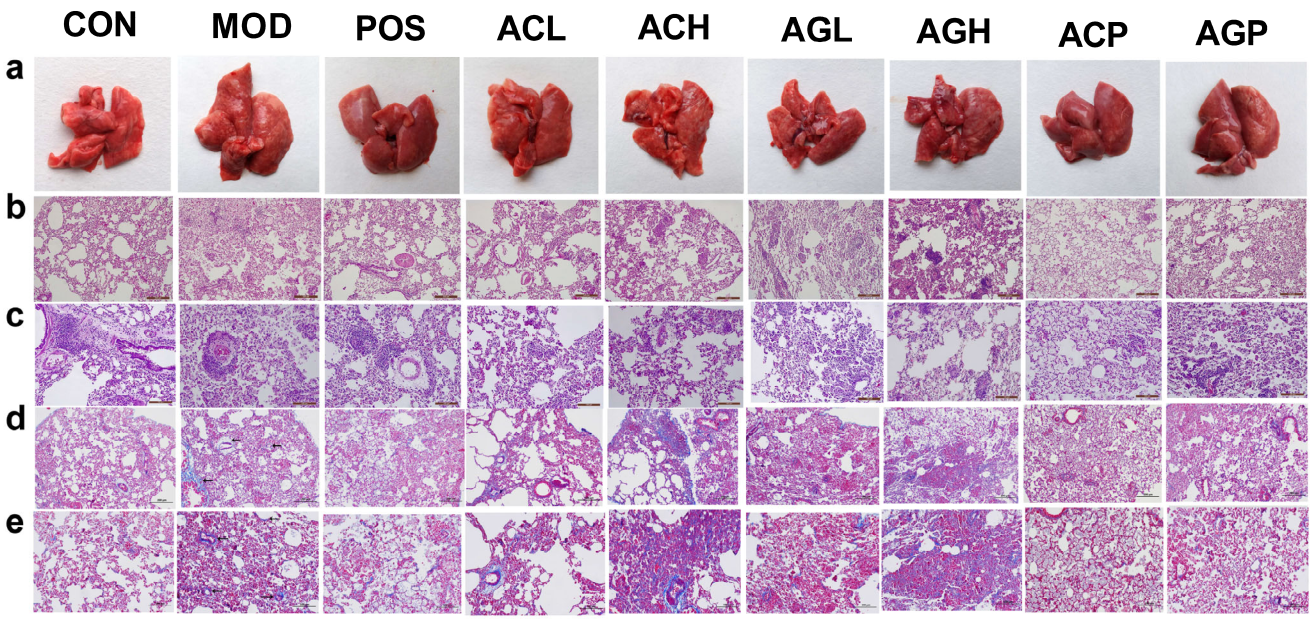

Figure S1 Lung tissues (a), H&E (b. 10×10, c. 20×10) and Masson (d. 10×10, e. 20×10) staining observation

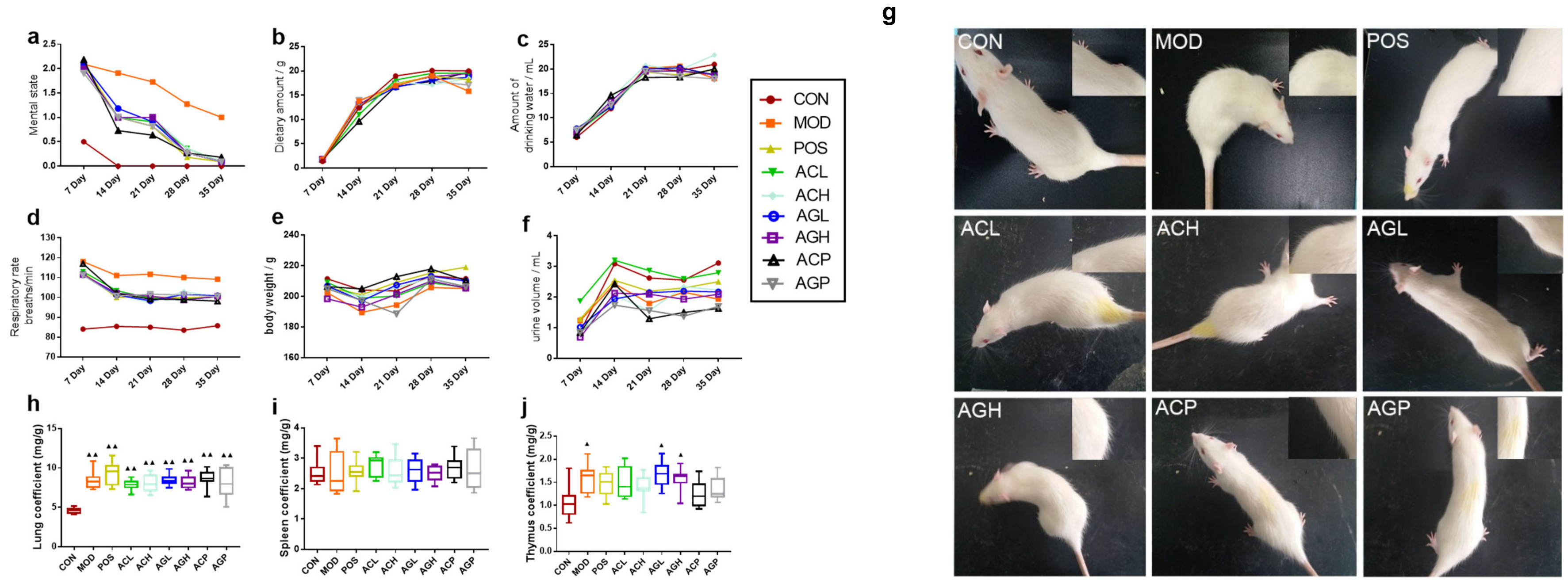

Figure S2 Mental state, body weight, fur luster, respiratory rate, urine volume, dietary amount and the amount of drinking water of rats (a-g). Organ coefficient (h. Lung coefficient, i. spleen coefficient, j. thymus coefficient) Note： mean ± SD, n = 8, ▲*P* <0.05, ▲▲*P* <0.01 *vs* CON； **P* <0.05, ***P* <0.01 *vs* MOD.

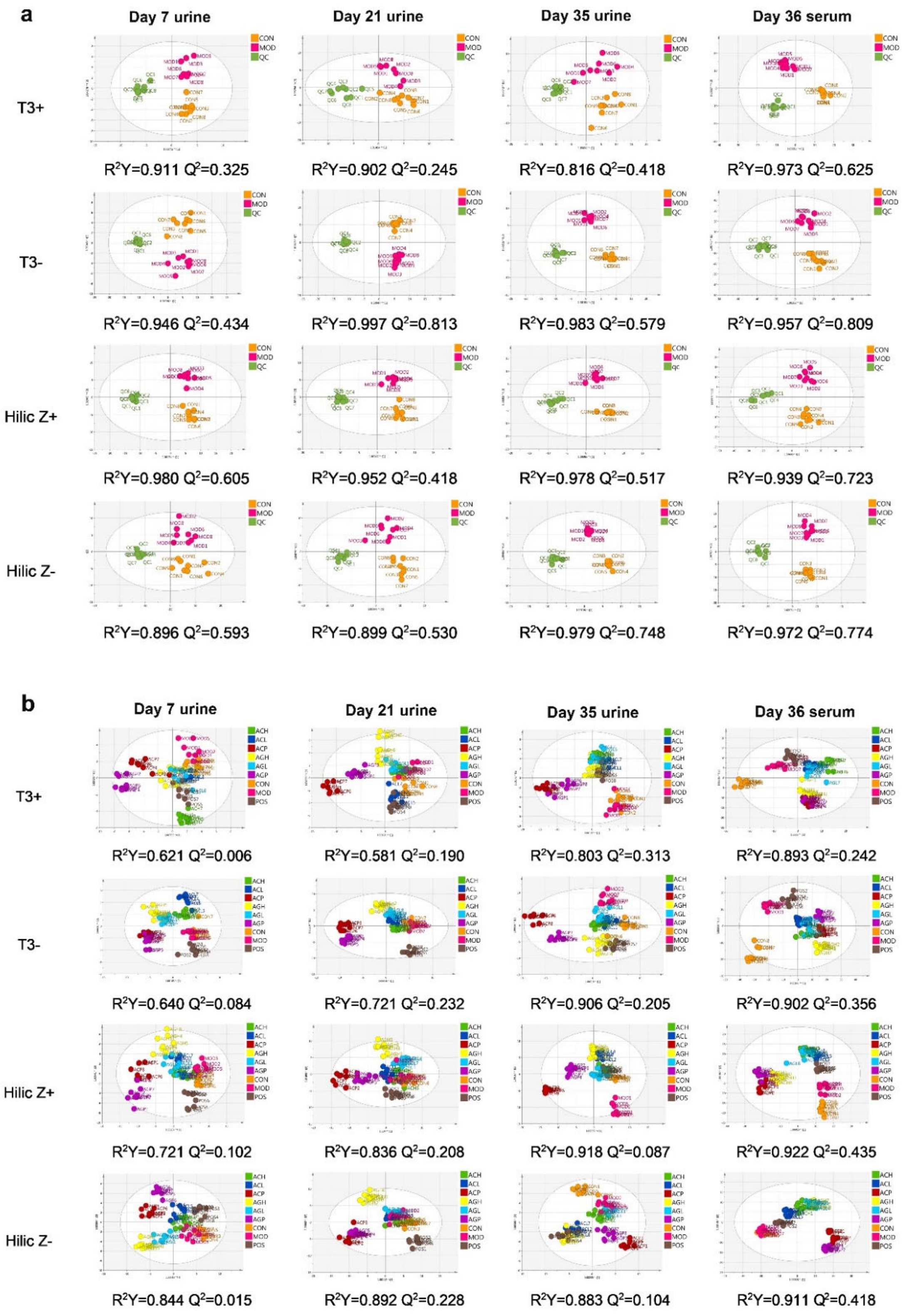

Figure S3 OPLS-DA plot of metabolomics (a. CON and MOD, b. All groups)

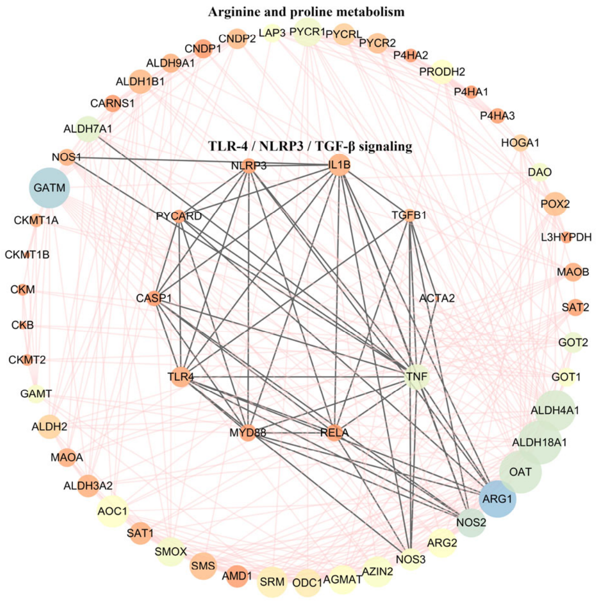

Figure S4 PPI network of related genes of argrine and proline metabolism and TLR-4 / NLRP3 /TGF-β signaling

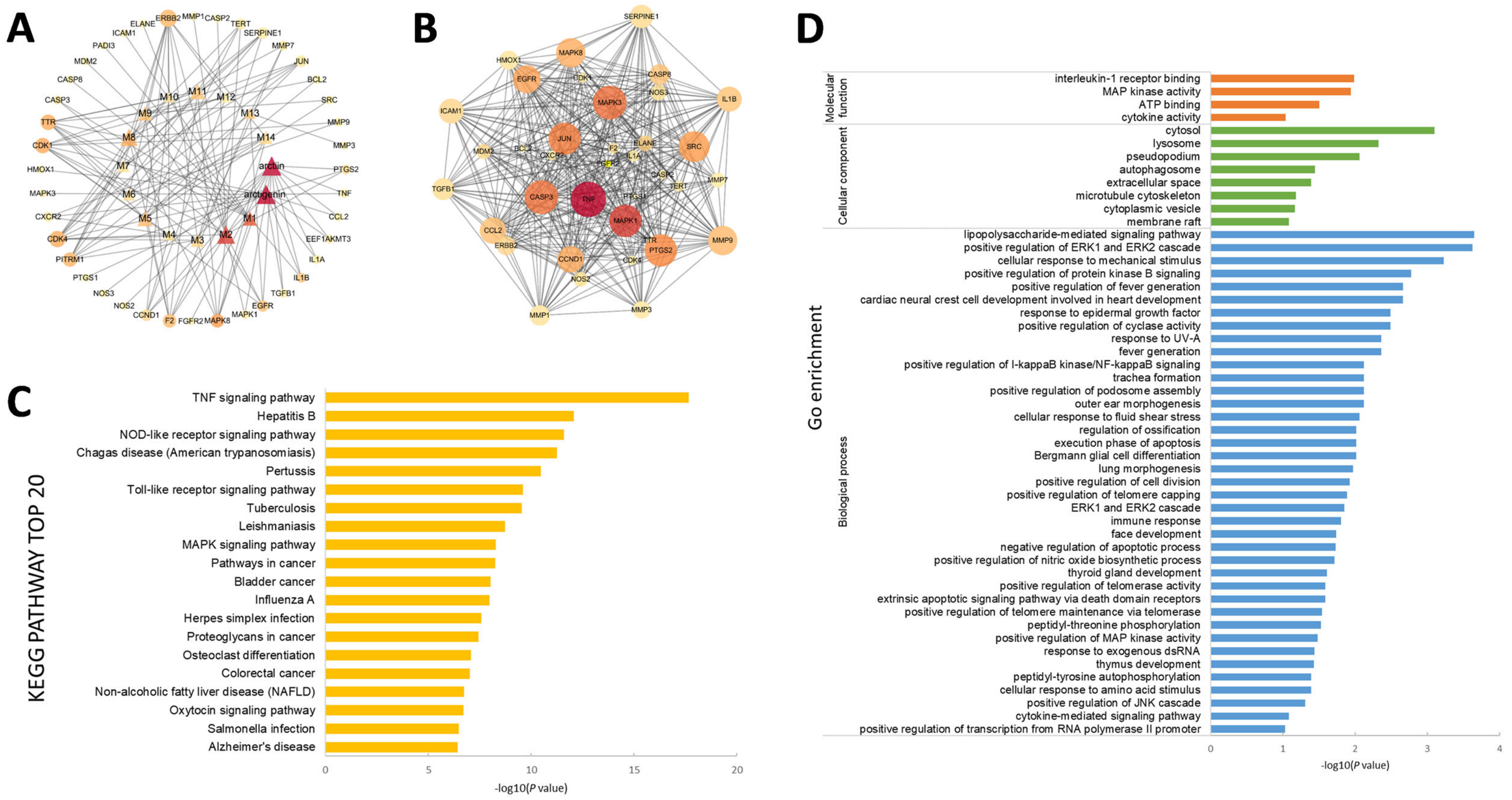

Figure S5 Arctiin and arctiin metabolites - target - PF interaction (A)，PPI network (B)， KEGG pathway (C) , GO enrichment (D)

Supplemental Table Legends

Table S1 Day 7 urine metabollites information. Notes: ★represent target metabolites, △represent differential metabolites in model, ○represent differential metabolites in arctiin, •represent differential metabolites in arctigenin. "-" represent metabolites with down-regulated trend, "+" represent metabolites with up-regulated trend, "--" and "++" represent significant difference from control at $P < 0.05$, "---" and "+++" represent significant difference from control at $P < 0.01$.

Table S2 Day21 urine metabollites information. Notes: ★represent target metabolites, △represent differential metabolites in model, ○represent differential metabolites in arctiin, •represent differential metabolites in arctigenin. "-" represent metabolites with down-regulated trend, "+" represent metabolites with up-regulated trend, "--" and "++" represent significant difference from control at $P < 0.05$, "---" and "+++" represent significant difference from control at $P < 0.01$.

Table S3 Day 35 urine metabollites information. Notes: ★represent target metabolites, △represent differential metabolites in model, ○represent differential metabolites in arctiin, •represent differential metabolites in arctigenin. "-" represent metabolites with down-regulated trend, "+" represent metabolites with up-regulated trend, "--" and "++" represent significant difference from control at $P < 0.05$, "---" and "+++" represent significant difference from control at $P < 0.01$.

Table S4 Day36 serum metabollites information. Notes: ★represent target metabolites, △represent differential metabolites in model, ○represent differential metabolites in arctiin, •represent differential metabolites in arctigenin. "-" represent metabolites with down-regulated trend, "+" represent metabolites with up-regulated trend, "--" and "++" represent significant difference from control at $P < 0.05$, "---" and "+++" represent significant difference from control at $P < 0.01$.

Table S5 Topological information of urine metabolites in various periods

Table S6 The importance of input nodes in modular network

Table S7 Oral bioavailability prediction of AdmetSAR2.0 web-survice

Table S8 XP Gscore of molecular docking studies

Table S1 Day 7 urine metabollites information.

HMDB	Ion mode	m/z	R.T. (min)	Mass	Formula	VIP	Compound Name	MOD	POS	ACL	ACH	AGL	AGH	ACP	AGP	Pathway
HMDB0000251	T3+	126.015	0.83	125.15	$C_2H_7NO_3S$	1.339	Taurine★	down	+	+	+	+	+	+	/	Taurine and hypotaurine metabolism; Primary bile acid biosynthesis
HMDB0000806	T3+	229.209	10.99	228.37	$C_{14}H_{28}O_2$	3.461	Myristic acid	up	-	-	-	-	-	-	-	/
HMDB0002820	T3+	141.059	1.10	140.14	$C_6H_8N_2O_2$	3.058	Methylimidazoleacetic acid△ ○●	down	+++	+	+	++	+++	++	++	/
HMDB0000860	T3+	208.090	3.35	207.23	$C_{11}H_{13}NO_3$	2.226	Phenylpropionylglycine△	up	-	-	-	-	/	/	/	/
HMDB0000701	T3+	174.106	3.46	173.2	$C_8H_{15}NO_3$	2.130	Hexanoylglycine△	up	-	-	-	-	-	-	/	/
HMDB0011663	T3+	181.043	2.91	180.16	$C_9H_8O_4$	0.764	3-(4-Hydroxyphenyl) pyruvate	down	+	+	/	+	+	+	++	Tyrosine metabolism; Phenylalanine, tyrosine and tryptophan biosynthesis; Ubiquinone and other terpenoid/quinone biosynthesis
HMDB0000268	T3+	351.246	4.92	350.49	$C_{21}H_{34}O_4$	0.484	Tetrahydrocorticosterone	up	/	-	-	-	-	/	/	Steroid hormone biosynthesis
HMDB0000101	T3+	252.102	0.94	251.24	$C_{10}H_{13}N_5O_3$	1.240	Deoxyadenosine	up	/	/	-	/	/	/	-	Purine metabolism
HMDB0004081	T3+	249.116	7.44	248.27	$C_{13}H_{16}N_2O_3$	1.143	6-Hydroxymelatonin	up	+	-	-	-	-	/	/	Tryptophan metabolism
HMDB0000210	T3-	218.111	3.55	219.23	$C_9H_{17}NO_5$	0.824	Pantothenic acid★○	up	/	--	-	-	/	/	/	Pantothenate and CoA biosynthesis
HMDB0000156	T3-	133.022	0.86	134.09	$C_4H_6O_5$	0.961	L-Malic acid★○●	up	-	--	--	--	-	-	-	Pyruvate metabolism; TCA cycle; Glyoxylate and dicarboxylate metabolism
HMDB0000357	T3-	103.030	3.83	104.10	C_4H_8OS	1.078	3-Hydroxybutyric acid★●	down	/	+	/	+	++	+	+	Butanoate metabolism; Synthesis and degradation of ketone bodies
HMDB0000517	T3-	173.11	3.54	174.20	$C_6H_{14}N_4O_2$	1.162	L-Arginine★	down	+	/	/	+	+	+	+	Arginine and proline metabolism; Aminoacyl/tRNA biosynthesis;Arginine biosynthesis
HMDB0003406	T3-	104.043	1.22	105.09	$C_3H_7NO_3$	0.801	D-Serine★	up	/	/	/	/	/	/	/	Glycine, serine and threonine metabolism
HMDB0000157	T3-	135.038	1.22	136.11	$C_5H_4N_4O$	1.489	Hypoxanthine●	down	/	+	/	++	+++	+++	++	Purine metabolism

HMDB0000309	T3-	181.044	1.22	182.14	$C_6H_6N_4O_3$	0.822	1-Methyluric acid●	down	/	+	/	+	++	++	/	Caffeine metabolism
HMDB0000459	T3-	156.074	3.71	157.17	$C_7H_{11}NO_3$	2.863	3-Methylcrotonylglycine△	up	-	-	-	-	/	/	-	/
HMDB0014511	T3-	311.209	9.5	312.45	$C_{21}H_{28}O_2$	2.848	Levonorgestrel△	down	++	+	+	+	/	+	+	/
HMDB0060014	T3-	269.074	4.37	270.24	$C_{12}H_{14}O_7$	2.277	Phenol glucuronide△○	down	+++	+	++	+	/	/	+	/
HMDB0000531	T3-	117.062	0.86	118.12	$C_5H_{10}O_3$	2.248	3-Hydroxyvaleric acid△○●	down	+	++	+	++	+	++	++	/
HMDB0000174	T3-	163.068	0.85	164.16	$C_6H_{12}O_5$	2.189	L-Fucose△○●	down	+	++	++	+++	+++	++	+++	/
HMDB0006372	T3-	105.027	0.81	106.08	$C_3H_6O_4$	2.078	L-Glyceric acid△	up	-	-	-	-	/	/	/	/
HMDB0000043	HILIC Z+	118.079	1.71	117.15	$C_5H_{11}NO_2$	0.691	Betaine★	down	+	+	+	+	/	/	/	Glycine, serine and threonine metabolism
HMDB0000073	HILIC Z+	154.081	0.5	153.18	$C_8H_{11}NO_2$	2.307	Dopamine★△	up	-	-	-	/	/	/	/	Tyrosine metabolism
HMDB0000452	HILIC Z+	104.064	1.99	103.12	$C_4H_9NO_2$	0.051	(S)-2-Aminobutanoic acid★	/	-	+	+	-	-	-	-	Cysteine and methionine metabolism
HMDB0000696	HILIC Z+	150.050	1.83	149.05	$C_5H_{11}NO_2S$	1.364	L-Methionine★	down	/	+	/	/	+	/	/	Cysteine and methionine metabolism; Aminoacyl/tRNA biosynthesis
HMDB0003411	HILIC Z+	116.063	0.93	115.13	$C_5H_9NO_2$	2.236	D-Proline★△●	up	/	-	-	-	---	---	---	Arginine and proline metabolism
HMDB0002284	HILIC Z+	145.126	1.62	144.21	$C_7H_{16}N_2O$	2.638	N-Acetylcadaverine△○●	up	-	---	-	-	--	--	-	/
HMDB0034708	HILIC Z+	183.131	4.24	182.26	$C_{11}H_{18}O_2$	2.568	Methyl 4,8-decadienoate△	down	/	/	/	/	+	+	/	/
HMDB0013816	HILIC Z+	205.152	1.59	206.33	$C_{14}H_{20}O$	2.436	2,4-Di-tert-butylphenol△	up	/	-	-	/	/	-	-	/
HMDB0002984	HILIC Z+	307.220	0.62	306.44	$C_{19}H_{30}O_3$	2.306	11-Hydroxyandrosterone△	up	---	-	-	-	-	/	/	/
HMDB0094793	HILIC Z+	198.105	2.24	197.23	$C_{10}H_{15}NO_3$	2.105	3,6-octadienoylglycine△	up	/	/	-	/	-	-	-	/
HMDB0001259	HILIC Z+	103.032	2.81	102.08	$C_4H_6O_3$	0.252	Succinic acid semialdehyde	/	/	+	+	+	+	+++	+++	Butanoate metabolism; Alanine, aspartate and glutamate metabolism
HMDB0000068	HILIC Z+	184.090	0.75	183.20	$C_9H_{13}NO_3$	0.578	Epinephrine●	/	-	-	-	-	--	---	---	Tyrosine metabolism
HMDB0000216	HILIC Z+	170.074	1.07	169.17	$C_8H_{11}NO_3$	1.330	Norepinephrine	down	-	/	/	-	/	-	/	Tyrosine metabolism
HMDB0011631	HILIC Z+	225.078	3.27	224.21	$C_{10}H_{12}N_2O_4$	1.524	L-3-Hydroxykynurenine	up	-	/	-	/	/	/	--	Tryptophan metabolism
HMDB0000472	HILIC Z+	221.086	1.94	220.22	$C_{11}H_{12}N_2O_3$	0.737	5-Hydroxy-L-tryptophan	up	-	/	-	+	+	+	-	Tryptophan metabolism
HMDB0001085	HILIC Z+	337.232	0.48	336.46	$C_{20}H_{32}O_4$	1.461	Leukotriene B4	down	-	-	-	-	-	-	-	Arachidonic acid metabolism
HMDB0000673	HILIC Z+	281.242	0.49	280.44	$C_{18}H_{32}O_2$	0.196	Linoleic acid	/	-	-	-	-	-	--	--	Linoleic acid metabolism;

																Biosynthesis of unsaturated fatty acids
HMDB0000190	HILIC Z-	89.031	2.43	90.08	$C_3H_6O_3$	0.551	L-Lactic acid★	down	+	/	/	/	/	/	/	Pyruvate metabolism
HMDB0000725	HILIC Z-	130.058	3.41	131.13	$C_5H_9NO_3$	0.299	4-Hydroxyproline★	/	-	/	-	--	-	/	-	Arginine and proline metabolism
HMDB0000763	HILIC Z-	190.058	0.64	191.18	$C_{10}H_9NO_3$	0.547	5-Hydroxyindoleacetic acid★ ●	/	-	-	/	+	++	++	/	Tryptophan metabolism
HMDB0000056	HILIC Z-	88.048	0.85	89.09	$C_3H_7NO_2$	1.209	beta-Alanine★	down	/	/	+	/	/	/	/	Pantothenate and CoA biosynthesis;<br>beta/Alanine metabolism;<br>Pyrimidine metabolism;<br>Propanoate metabolism
HMDB0001527	HILIC Z-	119.025	0.78	120.17	$C_4H_8O_2S$	0.243	3-Methylthiopropionic acid	/	/	/	+	+	+	+	+	Cysteine and methionine metabolism
HMDB0002820	HILIC Z-	139.058	1.83	140.13	$C_6H_8N_2O_2$	2.806	Methylimidazoleacetic acid△	down	++	+	+	+	+	++	++	Histidine metabolism
HMDB0000574	HILIC Z-	120.020	1.30	121.15	$C_3H_7NO_2S$	0.071	L-Cysteine	up	-	-	-	-	/	-	-	Pantothenate and CoA biosynthesis；<br>Cysteine and methionine metabolism;<br>Taurine and hypotaurine metabolism;<br>Glycine, serine and threonine metabolism;<br>Aminoacyl/tRNA biosynthesis;<br>Thiamine metabolism;<br>Glutathione metabolism
HMDB0003681	HILIC Z-	144.074	1.84	145.15	$C_6H_{11}NO_3$	2.543	4-Acetamidobutanoic acid△ ○●	down	+	++	++	++	+	+	+	Arginine and proline metabolism
HMDB0000227	HILIC Z-	147.074	1.05	148.15	$C_6H_{12}O_4$	1.663	Mevalonic acid○	down	/	-	--	+	+	/	-	Terpenoid backbone biosynthesis
HMDB0000479	HILIC Z-	168.085	2.39	169.18	$C_7H_{11}N_3O_2$	0.213	1-Methylhistidine	/	-	-	-	+	+	-	-	Histidine metabolism
HMDB0059727	HILIC Z-	173.089	1.03	174.19	$C_8H_{14}O_4$	2.949	2,4-Dimethyladipic acid△	up	-	-	-	-	/	/	-	/
HMDB0010313	HILIC Z-	313.064	1.18	314.24	$C_{13}H_{14}O_9$	3.082	1-Salicylate glucuronide△○	down	+	+	++	+	+	+	++	/
HMDB0031306	HILIC Z-	127.047	1.41	128.13	$C_6H_8O_3$	2.694	3-Hydroxy-4,5-dimethyl-2(5H)-furanone△ ○●	down	++	+	++	++	++	/	+	/

HMDB0059767	HILIC Z-	187.068	1.41	188.18	$C_8H_{12}O_5$	2.694	Tetrahydro-2,5-furan-diacetic acid△○●	down	++	+	++	++	++	/	+	Fatty acid biosynthesis
HMDB0000339	HILIC Z-	158.090	1.84	159.18	$C_7H_{13}NO_3$	2.655	2-Methylbutyrylglycine△○●	down	++	+++	+++	+++	+	+	+	/
HMDB0000343	HILIC Z-	285.158	0.47	286.37	$C_{18}H_{22}O_3$	2.549	2-Hydroxyestrone△	down	+	/	+	+	+	/	/	Steroid hormone biosynthesis
HMDB0000300	HILIC Z-	111.028	0.63	112.09	$C_4H_4N_2O_2$	1.968	Uracil△	up	/	-	-	-	-	-	-	Pantothenate and CoA biosynthesis;<br>beta/Alanine metabolism;<br>Pyrimidine metabolism
HMDB0000751	HILIC Z-	149.053	2.66	150.13	$C_5H_{10}O_5$	1.482	L-Xylulose△○	up	-	--	-	-	-	/	--	Pentose and glucuronate interconversions

Table S2 Day 21 urine metabollites information.

HMDB/KEGG	Ion mode	m/z	R.T. (min)	Mass	Formula	VIP	Compound Name	MOD	POS	ACL	ACH	AGL	AGH	ACP	AGP	Pathway
HMDB0000073	T3+	154.079	1.38	153.18	$C_8H_{11}NO_2$	0.349	Dopamine★	up	---	-	-	-	-	--	--	Tyrosine metabolism
HMDB0000195	T3+	269.081	3.43	268.23	$C_{10}H_{12}N_4O_5$	1.074	Inosine★	up	-	-	-	-	-	---	---	Purine metabolism
HMDB0000251	T3+	126.015	0.85	125.15	$C_2H_7NO_3S$	1.051	Taurine★○	up	/	-	--	-	-	-	-	Taurine and hypotaurine metabolism；<br>Primary bile acid biosynthesis
HMDB0000291	T3+	199.053	0.81	198.17	$C_9H_{10}O_5$	2.180	4-Hydroxy-3-methoxymandelic acid★△○	down	+	+	+ +	+	+	+ + +	+ +	Tyrosine metabolism
HMDB0000574	T3+	122.019	3.99	121.15	$C_3H_7NO_2S$	1.332	L-Cysteine	up	/	+	/	-	/	+	+	Pantothenate and CoA biosynthesis；<br>Taurine and hypotaurine metabolism；<br>Cysteine and methionine metabolism；<br>Thiamine metabolism；<br>Glutathione metabolism；<br>Aminoacyl-tRNA biosynthesis；<br>Glycine, serine and threonine metabolism
HMDB0004081	T3+	249.116	7.51	248.27	$C_{13}H_{16}N_2O_3$	2.204	6-Hydroxymelatonin△	down	+	/	/	/	/	/	/	/

HMDB0000299	T3+	285.076	3.16	284.22	$C_{10}H_{12}N_4O_6$	0.572	Xanthosine	up	-	-	-	-	-	-	-	Purine metabolism
HMDB0000259	T3+	177.095	3.12	176.22	$C_{10}H_{12}N_2O$	3.228	Serotonin△	up	- - -	-	-	-	-	- -	- -	Tryptophan metabolism
HMDB0000123	T3-	74.032	4.22	75.07	$C_2H_5NO_2$	0.253	Glycine★	/	-	/	+	+	+	-	---	Primary bile acid biosynthesis；<br>Glutathione metabolism；<br>Aminoacyl-tRNA biosynthesis；<br>Glycine, serine and threonine metabolism；<br>Porphyrin and chlorophyll metabolism；<br>Glyoxylate and dicarboxylate metabolism
HMDB0000210	T3-	218.11	3.23	219.23	$C_9H_{17}NO_5$	1.409	Pantothenic acid★△	up	-	-	/	-	/	/	/	Pantothenate and CoA biosynthesis
HMDB0000619	T3-	407.287	7.69	408.57	$C_{24}H_{40}O_5$	0.095	Cholic acid★	/	-	+	-	-	+	/	/	Primary bile acid biosynthesis
HMDB0000309	T3-	181.044	1.3	182.14	$C_6H_6N_4O_3$	0.092	1-Methyluric acid	/	-	-	-	+	+	+	+	Caffeine metabolism
HMDB0000593	T3-	784.596	11.03	785.11	$C_{44}H_{84}NO_8P$	1.189	PC(18:1(9Z)/18:1(9Z)) ○●	up	--	-	--	-	---	---	--	Linoleic acid metabolism；<br>Arachidonic acid metabolism；<br>alpha-Linolenic acid metabolism；<br>Glycerophospholipid metabolism
HMDB0008591	T3-	782.578	0.63	783.09	$C_{44}H_{82}NO_8P$	1.404	PC(22:2(13Z,16Z)/14:1(9Z))	up	-	-	/	+	+	+	+	Linoleic acid metabolism；<br>Arachidonic acid metabolism；<br>Alpha-inolenic acid metabolism；<br>Glycerophospholipid metabolism
HMDB0000300	T3-	111.026	1.07	112.08	$C_4H_4N_2O_2$	0.063	Uracil	/	-	+	+	+	+	+	+	Pantothenate and CoA biosynthesis；<br>beta-Alanine metabolism；<br>Pyrimidine metabolism
HMDB0000068	T3-	182.09	4.35	183.2	$C_9H_{13}NO_3$	0.924	Epinephrine	up	/	-	-	-	/	--	--	Tyrosine metabolism
HMDB0000347	T3-	287.172	6.46	288.38	$C_{18}H_{24}O_3$	2.321	16b-Hydroxyestradiol△ ○●	up	- - -	-	- -	- -	-	- - -	- - -	/
HMDB0059731	T3-	155.042	2.83	156.14	$C_7H_8O_4$	1.935	2,3-Methyleneglutaric acid△ ○●	down	+	+ +	+	+ +	+ +<br>+	+	+ +	/

HMDB0000673	T3-	279.24	9.42	280.45	$C_{18}H_{32}O_2$	2.000	Linoleic acid△	up	-	/	-	-	/	-	-	Linoleic acid metabolism
HMDB0000531	T3-	117.063	2.92	118.13	$C_5H_{10}O_3$	1.475	3-Hydroxyvaleric acid△○●	down	/	+ + +	+	+	+ +	+ + +	+ +	/
HMDB0002511	T3-	237.084	6.29	238.24	$C_{12}H_{14}O_5$	1.683	3,4,5-Trimethoxycinnamic acid△	down	/	/	/	/	/	+	/	/
HMDB0000909	T3-	157.094	6.58	158.2	$C_8H_{14}O_3$	1.856	trans-4-Hydroxycyclohexylacetic acid△	down	+	+	+	+	+	/	/	/
HMDB0000452	HILIC Z+	104.064	2.01	103.12	$C_4H_9NO_2$	1.834	(S)-2-Aminobutanoate★●	up	-	-	-	/	--	-	-	Cysteine and methionine metabolism
HMDB0000696	HILIC Z+	150.051	1.87	149.05	$C_5H_{11}NO_2S$	0.545	L-Methionine★	up	-	/	/	/	+	+	+	Cysteine and methionine metabolism；Aminoacyl-tRNA biosynthesis
HMDB0011631	HILIC Z+	225.079	3.28	224.21	$C_{10}H_{12}N_2O_4$	0.819	L-3-Hydroxykynurenine	down	+	+	+	-	+	+	+	Tryptophan metabolism
HMDB0001259	HILIC Z+	103.032	2.84	102.08	$C_4H_6O_3$	0.241	Succinic acid semialdehyde	/	+	+	/	/	+	+	+	Butanoate metabolism；Alanine, aspartate and glutamate metabolism
HMDB0000289	HILIC Z+	169.028	1.51	168.11	$C_5H_4N4O_3$	0.857	Uric acid	/	+	-	+	+	-	--	---	Purine metabolism
HMDB0000216	HILIC Z+	170.075	1.57	169.17	$C_8H_{11}NO_3$	0.849	Norepinephrine●	/	+	+	+	+	+++	/	/	Tyrosine metabolism
HMDB0000472	HILIC Z+	221.085	1.96	220.22	$C_{11}H_{12}N_2O_3$	0.711	5-Hydroxy-L-tryptophan	up	-	+	-	+	+	+	+	Tryptophan metabolism
HMDB0001085	HILIC Z+	337.232	0.48	336.46	$C_{20}H_{32}O_4$	0.874	Leukotriene B4	up	-	-	-	-	-	---	---	Arachidonic acid metabolism
HMDB0004827	HILIC Z+	144.095	1.6	143.18	$C_7H_{13}NO_2$	1.770	Proline betaine△	down	/	/	/	/	/	/	/	/
HMDB0170858	HILIC Z+	101.053	2.98	100.12	$C_5H_8O_2$	2.588	3-Methylbut-3-enoic acid△○●	up	-	- - -	- -	-	- - -	-	-	/
HMDB0000806	HILIC Z+	229.209	0.3	228.37	$C_{14}H_{28}O_2$	1.509	Myristic acid△●	up	/	/	/	-	- -	-	-	/
HMDB0094806	HILIC Z+	200.121	2.96	199.25	$C_{10}H_{17}NO_3$	1.841	7-octenoylglycine△	down	+	+	+	+	+	+	+	/
HMDB0094793	HILIC Z+	198.107	4.16	197.23	$C_{10}H_{15}NO_3$	1.946	3,6-octadienoylglycine△	down	+ +	+	+	+	+	+	+	/
HMDB0000357	HILIC Z-	103.047	2.49	104.1	$C_4H_8O_3$	1.501	3-Hydroxybutyric acid★△	down	/	/	/	/	/	/	/	Butanoate metabolism；Synthesis and degradation of ketone bodies

HMDB0000763	HILIC Z-	190.058	0.63	191.18	$C_{10}H_9NO_3$	0.628	5-Hydroxyindoleacetic acid★	/	-	-	/	/	+	+	+	Tryptophan metabolism
HMDB0000725	HILIC Z-	130.058	3.48	131.13	$C_5H_9NO_3$	1.119	4-Hydroxyproline★	up	---	-	-	-	-	-	-	Arginine and proline metabolism
HMDB0000056	HILIC Z-	88.048	0.86	89.09	$C_3H_7NO_2$	1.512	beta-Alanine★●	/	-	-	/	-	--	--	---	Pantothenate and CoA biosynthesis；beta-Alanine metabolism；Pyrimidine metabolism；Propanoate metabolism
HMDB0000562	HILIC Z-	114.063	1.39	115.13	$C_5H_9NO_2$	0.657	D-Proline★	/	-	/	+	-	/	+	+	Arginine and proline metabolism
HMDB0000673	HILIC Z-	279.24	0.43	280.44	$C_{18}H_{32}O_2$	0.166	Linoleic acid●	up	-	+	-	+	++	-	+	Linoleic acid metabolism；Biosynthesis of unsaturated fatty acids
HMDB0004073	HILIC Z-	174.063	1.23	175.18	$C_{10}H_9N_{O2}$	0.097	5-Hydroxyindoleacetaldehyde	/	-	/	/	/	+	+	+	Tryptophan metabolism
HMDB0000132	HILIC Z-	150.05	1.16	151.12	$C_5H_5N_5O$	0.949	Guanine	up	-	-	-	-	-	-	/	Purine metabolism
HMDB0001139	HILIC Z-	353.241	0.49	354.48	$C_{20}H_{34}O_5$	0.228	Prostaglandin F2a	up	+	-	-	+	+	+	+	Arachidonic acid metabolism
HMDB0000071	HILIC Z-	251.086	2.81	252.23	$C_{10}H_{12}N_4O_4$	1.667	Deoxyinosine	down	-	+	+	+	+	+	/	Purine metabolism
HMDB0002820	HILIC Z-	139.058	1.88	140.13	$C_6H_8N_2O_2$	1.278	Methylimidazoleacetic acid	down	+	-	-	/	/	+	+	Histidine metabolism
HMDB0003681	HILIC Z-	144.074	1.91	145.15	$C_6H_{11}NO_3$	2.521	4-Acetamidobutanoic acid△	up	-	-	-	-	/	-	- - -	Arginine and proline metabolism
HMDB0000227	HILIC Z-	147.074	1.03	148.15	$C_6H_{12}O_4$	0.021	Mevalonic acid○	/	+	++	+	+	+	++	++	Terpenoid backbone biosynthesis
HMDB0034520	HILIC Z-	645.372	1.15	646.8	$C_{36}H_{54}O_{10}$	0.589	Gypsogenin 3-O-β-D-glucuronide	up	-	-	-	-	/	+++	++	/
HMDB0000606	HILIC Z-	147.037	1.46	148.11	$C_5H_8O_5$	1.067	2-Hydroxyglutarate	down	-	+	+	-	+	+	+	Butanoate metabolism
HMDB0000479	HILIC Z-	168.085	2.51	169.18	$C_7H_{11}N_3O_2$	0.004	1-Methylhistidine	/	-	-	-	/	-	+	+	Histidine metabolism
HMDB0000725	HILIC Z-	216.988	1.05	218.18	$C_7H_6O_6S$	2.303	4-hydroxybenzoic acid-4-O-sulphate△	up	- -	-	-	/	/	/	/	/
HMDB0000603	HILIC Z-	199.105	0.53	200.23	$C_{10}H_{16}O_4$	2.132	cis-4-Decenedioic acid△	up	- - -	/	/	/	-	-	-	/
HMDB0000751	HILIC Z-	149.053	2.48	150.13	$C_5H_{10}O_5$	1.755	L-Xylulose△	down	/	/	/	/	/	/	/	Pentose and glucuronate interconversions

HMDB0011723	HILIC Z-	192.074	1.47	193.2	$C_{10}H_{11}NO_3$	1.864	2-Methylhippuric acid△	up	-	-	-	-	-	-	-	/
HMDB0000418	HILIC Z-	377.204	0.86	378.46	$C_{21}H_{30}O_6$	1.716	18-Hydroxycortisol△	down	/	/	/	/	/	/	/	/
HMDB0000620	HILIC Z-	129.026	2.56	130.1	$C_5H_6O_4$	1.748	Glutaconic acid△	up	-	-	-	/	-	/	-	/
HMDB0000508	HILIC Z-	151.068	2.3	152.15	$C_5H_{12}O_5$	2.161	Ribitol△●	up	-	-	-	-	- -	-	-	/
HMDB0000845	HILIC Z-	252.082	2.81	253.21	$C_9H_{11}N_5O_4$	2.180	Neopterin△	up	-	-	-	-	-	-	- -	/

Table S3 Day 35 urine metabollites information.

HMDB	Ion mode	m/z	R.T. (min)	Mass	Formula	VIP	Compound Name	MOD	POS	ACL	ACH	AGL	AGH	ACP	AGP	Pathway
HMDB0000210	T3+	220.111	3.57	219.23	$C_9H_{17}NO_5$	0.528	Pantothenic acid★	/	-	-	-	-	-	-	-	Pantothenate and CoA biosynthesis
HMDB0000517	T3+	175.112	4.83	174.20	$C_6H_{14}N_4O_2$	0.423	L-Arginine★	/	+	/	/	/	/	++	+	Arginine biosynthesis; Arginine and proline metabolism; Aminoacyl/tRNA biosynthesis
HMDB0000268	T3+	351.246	4.82	350.49	$C_{21}H_{34}O_4$	1.704	Tetrahydrocorticosterone△	up	-	-	/	/	/	/	/	Steroid hormone biosynthesis
HMDB0001259	T3+	103.032	4.09	102.08	$C_4H_6O_3$	0.327	Succinic acid semialdehyde●	/	+	/	+	++	++	+++	+++	Butanoate metabolism; Alanine, aspartate and glutamate metabolism
HMDB0001851	T3+	153.067	4.42	152.14	$C_5H_{12}O_5$	1.759	L-Arabitol△	up	/	/	/	/	/	/	/	Pentose and glucuronate interconversions
HMDB0000630	T3+	112.043	0.86	111.1	$C_4H_5N_3O$	2.558	Cytosine△○●	down	++	+++	+++	+++	+++	+++	+++	/
HMDB0002064	T3+	131.111	3.36	166.65	$C_6H_{14}N_2O$	2.047	N-Acetylputrescine△●	down	+	+	/	+	++	+	+	Arginine and proline metabolism
HMDB0011309	T3+	770.598	5.64	770.11	$C_{44}H_{84}NO_7P$	1.655	PC(P-18:1(9Z)/18:1(9Z)) △●	down	/	-	-	---	/	---	/	/
HMDB0000357	T3-	103.047	0.79	104.10	$C_4H_8O_3$	1.943	3-Hydroxybutyric acid★△	down	/	+	+	+	/	/	/	Butanoate metabolism; Synthesis and degradation of ketone bodies
HMDB0000123	T3-	74.032	4.26	75.07	$C_2H_5NO_2$	2.383	Glycine★△	up	-	-	-	-	-	---	-	Glutathione metabolism; Porphyrin and chlorophyll metabolism; Aminoacyl/tRNA biosynthesis; Glyoxylate and dicarboxylate metabolism;

HMDB0000300	T3-	111.027	2.81	112.08	$C_4H_4N_2O_2$	1.161	Uracil●	up	+	-	-	--	-	/	-	Glycine, serine and threonine metabolism; Primary bile acid biosynthesis Pantothenate and CoA biosynthesis; beta/Alanine metabolism; Pyrimidine metabolism
HMDB0000068	T3-	182.09	4.37	183.2	$C_9H_{13}NO_3$	0.055	Epinephrine●	/	+	-	-	--	-	---	-	Tyrosine metabolism
HMDB0000157	T3-	135.038	1.39	136.11	$C_5H_4N_4O$	0.113	Hypoxanthine	/	+	-	+	+	-	/	-	Purine metabolism
HMDB0003357	T3-	173.099	1.5	174.19	$C_7H_{14}N_2O_3$	0.622	N-Acetylornithine	/	--	-	+	-	+	+	-	Arginine biosynthesis
HMDB0000214	T3-	131.09	1.5	132.16	$C_5H_{12}N_2O_2$	0.987	Ornithine	/	-	-	-	-	-	/	-	Arginine biosynthesis; Arginine and proline metabolism; Sulfur metabolism
HMDB0003099	T3-	181.044	1.41	182.14	$C_6H_6N_4O_3$	1.723	1-Methyluric acid△●	up	-	-	-	-	--	-	--	Caffeine metabolism
HMDB0094793	T3-	196.105	6.03	197.23	$C_{10}H_{15}NO_3$	1.828	3,6-octadienoylglycine△○●	up	-	--	--	--	-	---	---	/
HMDB0059975	T3-	289.046	4.48	290.29	$C_{11}H_{14}O_7S$	1.677	4-Hydroxy-5-(3'-hydroxyphenyl)- valeric acid-3'-O-sulphate△	up	--	-	/	/	-	/	-	/
HMDB0000806	T3-	227.209	7.06	228.37	$C_{14}H_{28}O_2$	2.133	Myristic acid△○●	up	---	--	---	--	-	--	--	/
HMDB0013816	T3-	203.151	7.26	206.33	$C_{14}H_{20}O$	1.921	2,4-Di-tert-butylphenol△●	up	-	---	--	---	-	-	-	/
HMDB0000063	T3-	361.209	7.48	362.46	$C_{21}H_{30}O_5$	1.927	Cortisol△●	up	---	-	-	---	--	-	-	Steroid hormone biosynthesis
HMDB0001861	HILIC Z+	126.095	3.49	125.17	$C_6H_{11}N_3$	2.708	3-Methylhistamine△○●	up	---	---	--	---	---	---	---	/
HMDB0002277	HILIC Z+	343.204	0.49	342.43	$C_{18}H_{30}O_6$	1.822	2,3-Dinor-6-keto-prostaglandin F1 a△○●	up	---	---	-	---	---	-	-	/
HMDB0011253	HILIC Z+	794.598	2.08	794.14	$C_{46}H_{84}NO_7P$	2.041	PC(P-18:0/20:4(5Z,8Z,11Z,14Z)) △ ○●	up	-	-	--	-	---	-	-	/

HMDB0059768	HILIC Z+	199.089	5.32	198.22	$C_{10}H_{14}O_4$	2.108	3,4-Methylene suberic acid△○●	up	--	--	---	--	--	--	---	/
HMDB0004073	HILIC Z+	176.063	1.99	175.18	$C_{10}H_9NO_2$	1.761	5-Hydroxyindoleacetaldehyde △○	up	-	--	--	-	-	-	-	Tryptophan metabolism
HMDB0000339	HILIC Z+	160.09	4.99	159.18	$C_7H_{13}NO_3$	1.495	2-Methylbutyrylglycine△●	up	-	-	-	---	-	-	-	/
HMDB0000022	HILIC Z+	168.095	5.24	167.21	$C_9H_{13}NO_2$	1.685	3-Methoxytyramine△○	up	-	--	-	-	-	-	-	Tyrosine metabolism
HMDB0061384	HILIC Z+	185.121	5.24	184.24	$C_9H_{16}N_2O_2$	1.685	N-(3-acetamidopropyl)pyrrolidin-2-one△○	up	-	--	-	-	-	-	-	/
HMDB0010313	HILIC Z+	315.064	3.76	314.25	$C_{13}H_{14}O_9$	1.926	1-Salicylate glucuronide△○	up	-	-	--	-	-	-	-	/
HMDB0000195	HILIC Z-	267.08	4.12	268.23	$C_{10}H_{12}N_4O_5$	0.186	Inosine★	/	-	-	/	-	+	-	-	Purine metabolism
HMDB0000725	HILIC Z-	130.058	2.82	131.13	$C_5H_9NO_3$	1.106	4-Hydroxyproline★	up	/	/	/	/	/	/	/	Arginine and proline metabolism
HMDB0000227	HILIC Z-	147.074	1.55	148.15	$C_6H_{12}O_4$	0.189	Mevalonic acid	/	/	/	+	+	+	+++	+++	Terpenoid backbone biosynthesis
HMDB0034520	HILIC Z-	645.374	1.23	646.8	$C_{36}H_{54}O_{10}$	0.167	Gypsogenin 3-O-β-D-glucuronide	/	-	+	+	+	/	+++	+++	Pentose and glucuronate interconversions
HMDB0000024	HILIC Z-	890.647	2.36	891.65	$C_{48}H_{93}NO_{11}S$	0.750	3-O-Sulfogalactosylceramide (d18:1/24:0)○●	/	---	---	---	---	---	---	---	Sphingolipid metabolism
HMDB0000054	HILIC Z-	583.266	1.25	584.66	$C_{33}H_{36}N_4O_6$	1.096	Bilirubin	up	-	/	+	-	-	++	+	Porphyrin and chlorophyll metabolism
HMDB0001644	HILIC Z-	149.053	2.55	150.12	$C_5H_{10}O_5$	0.709	D-Xylulose●	/	-	-	-	-	---	-	-	Pentose and glucuronate interconversions
HMDB0059655	HILIC Z-	147.038	1.65	148.11	$C_5H_8O_5$	1.042	2-Hydroxyglutaric acid	up	-	-	/	/	-	-	/	Butanoate metabolism
HMDB0000001	HILIC Z-	168.085	4.96	169.18	$C_7H_{11}N_3O_2$	1.148	1-Methylhistidine	up	-	-	/	-	-	-	+	Histidine metabolism
HMDB0001139	HILIC Z-	353.24	0.49	354.48	$C_{20}H_{34}O_5$	0.864	Prostaglandin F2a	up	-	-	/	/	-	/	/	Arachidonic acid metabolism
HMDB0000071	HILIC Z-	251.086	3.01	252.23	$C_{10}H_{12}N_4O_4$	0.443	Deoxyinosine	up	-	-	/	/	-	-	-	Purine metabolism
HMDB0059731	HILIC Z-	155.042	0.9	156.14	$C_7H_8O_4$	2.751	2,3-Methyleneglutaric acid△●	down	/	+	+	++	++	+	+	/
HMDB0059982	HILIC Z-	216.989	1.17	218.18	$C_7H_6O_6S$	2.025	4-hydroxybenzoic acid-4-O-sulphate△○●	up	--	--	-	-	---	-	-	/

HMDB0000459	HILIC Z-	156.074	1.51	157.17	$C_7H_{11}NO_3$	2.039	3-Methylcrotonylglycine△	up	-	/	/	/	-	/	/	/
HMDB0000965	HILIC Z-	108.02	2.59	109.15	$C_2H_7NO_2S$	1.961	Hypotaurine△○●	up	---	-	---	---	--	---	---	Taurine and hypotaurine metabolism
HMDB0000701	HILIC Z-	172.105	0.95	173.21	$C_8H_{15}NO_3$	0.700	Hexanoylglycine△	down	/	/	+	+	/	+	/	/
HMDB0004827	HILIC Z-	142.095	1.42	143.18	$C_7H_{13}NO_2$	1.303	Proline betaine△	up	-	-	-	-	-	-	-	/
HMDB0006049	HILIC Z-	260.041	1.68	261.17	$C_9H_{12}NO_6P$	1.709	O-Phosphotyrosine△●	up	-	-	-	--	-	-	-	/
HMDB0170858	HILIC Z-	99.053	1.44	100.11	$C_5H_8O_2$	1.449	3-Methylbut-3-enoic acid△	up	-	/	/	/	/	/	/	/
HMDB0000660	HILIC Z-	179.064	3.11	180.16	$C_6H_{12}O_6$	1.733	D-Fructose△	down	+	+	+	+	+	/	+	Amino sugar and nucleotide sugar metabolism
HMDB0033742	HILIC Z-	101.068	1.01	102.13	$C_5H_{10}O_2$	1.289	(S)-2-Methylbutanoic acid△	down	/	/	+	+	/	+	+	/
HMDB0000205	HILIC Z-	163.047	0.91	164.16	$C_9H_8O_3$	1.208	Phenylpyruvic acid△	down	/	+	+	+	/	+	+	Phenylalanine, tyrosine and tryptophan biosynthesis;<br>Phenylalanine metabolism

Table S4 Day36 serum metabollites information.

HMDB	Ion mode	m/z	R.T. (min)	Mass	Formula	VIP	Compound Name	MOD	POS	ACL	ACH	AGL	AGH	ACP	AGP	Pathway
HMDB0001043	T3(ESI+)	305.24	10.3	304.47	$C_{20}H_{32}O_2$	0.872	Arachidonic acid★○●	up	++	+++	+++	+++	+++	+++	+++	Arachidonic acid metabolism；<br>Biosynthesis of unsaturated fatty acids
HMDB0000182	T3(ESI+)	147.105	0.59	146.19	$C_6H_{14}N_2O_2$	1.068	L-Lysine★	down	++	+	+	+	+	+	++	Aminoacyl-tRNA biosynthesis；<br>Biotin metabolism；Lysine degradation
HMDB0000222	T3(ESI+)	400.335	8.49	399.61	$C_{23}H_{45}NO_4$	2.240	L-Palmitoylcarnitine△●	down	/	/	/	+	+++	++	+	Fatty acid degradation
HMDB0010403	T3(ESI+)	570.348	8.60	569.71	$C_{30}H_{52}NO_7P$	1.185	LysoPC(22:5(7Z,10Z,13Z,16Z,19Z)) ○●	up	/	++	-	+++	+++	+	++	Linoleic acid metabolism;<br>Arachidonic acid metabolism；<br>Glycerophospholipid metabolism；<br>alpha-Linolenic acid metabolism
HMDB0001257	T3(ESI+)	146.158	0.58	145.25	$C_7H_{19}N_3$	1.698	Spermidine△	down	+	/	/	+	+	+	/	Arginine and proline metabolism；

																Histidine metabolism；beta-Alanine metabolism；Glutathione metabolism
HMDB0000138	T3(ESI+)	466.309	6.18	465.62	$C_{26}H_{43}NO_6$	0.271	Glycocholic acid	down	--	-	-	-	-	-	-	Primary bile acid biosynthesis
HMDB0002088	T3(ESI+)	326.298	8.02	325.53	$C_{20}H_{39}NO_2$	2.187	N-Oleoylethanolamine△○●	down	/	+	+++	++	+++	+++	+++	/
HMDB0060012	T3(ESI+)	333.183	6.91	332.39	$C_{16}H_{28}O_7$	2.319	Neomenthol-glucuronide△	down	+	/	+	+	+	+	+	/
HMDB0001173	T3(ESI+)	298.09	2.81	297.33	$C_{11}H_{15}N_5O_3S$	1.733	5'-Methylthioadenosine△	down	/	-	-	/	/	-	/	Cysteine and methionine metabolism
HMDB0000043	T3(ESI-)	116.079	0.87	117.15	$C_5H_{11}NO_2$	0.993	Betaine★●	down	+	+	+	+	++	+	+	Glycine, serine and threonine metabolism
HMDB0000254	T3(ESI-)	117.027	1.24	118.08	$C_4H_6O_4$	1.453	Succinate★△	down	/	+	+	+	+	+	/	Butanoate metabolism;<br>Citrate cycle (TCA cycle)；<br>Alanine, aspartate and glutamate metabolism；<br>Propanoate metabolism
HMDB0000094	T3(ESI-)	191.027	1.07	192.12	$C_6H_8O_7$	0.458	Citrate★	up	+	+	-	+	+	+	-	Alanine, aspartate and glutamate metabolism；<br>Citrate cycle (TCA cycle)；<br>Glyoxylate and dicarboxylate metabolism
HMDB0000827	T3(ESI-)	283.271	9.46	284.48	$C_{18}H_{36}O_2$	1.270	Stearic acid★△	up	/	/	-	-	-	-	-	/
HMDB0000251	T3(ESI-)	124.015	0.70	125.15	$C_2H_7NO_3S$	1.008	Taurine★△○	down	+++	++	+	+	+	/	+	Taurine and hypotaurine metabolism；<br>Primary bile acid biosynthesis
HMDB0000357	T3(ESI-)	103.047	1.62	104.1	$C_4H_8O_3$	0.253	(R)-3-Hydroxybutanoate★●	down	+	+	+	/	++	++	++	Butanoate metabolism；<br>Synthesis and degradation of ketone bodies
HMDB0000148	T3(ESI-)	146.053	0.74	147.13	$C_5H_9NO_4$	0.824	L-Glutamic acid★△	up	/	/	/	/	-	/	-	Butanoate metabolism；<br>Arginine and proline metabolism；<br>Aminoacyl-tRNA biosynthesis；<br>D-Glutamine and D-glutamate metabolism；<br>Alanine, aspartate and glutamate metabolism；<br>Arginine biosynthesis；Histidine metabolism；<br>Nitrogen metabolism；Glutathione metabolism；

HMDB0000177	T3(ESI-)	154.069	0.66	155.15	$C_6H_9N_3O_2$	0.965	L-Histidine★	up	++	-	/	+	+	+	+	Glyoxylate and dicarboxylate metabolism；Glycine, serine and threonine metabolism；Porphyrin and chlorophyll metabolism Aminoacyl-tRNA biosynthesis；Histidine metabolism；beta-Alanine metabolism
HMDB0000159	T3(ESI-)	180.074	1.21	181.19	$C_9H_{11}NO_3$	0.314	L-Tyrosine★○	down	/	--	-	-	-	-	---	Aminoacyl-tRNA biosynthesis；Phenylalanine, tyrosine and tryptophan biosynthesis；Phenylalanine metabolism；Tyrosine metabolism；Ubiquinone and other terpenoid-quinone biosynthesis；
HMDB0001220	T3(ESI-)	351.224	7.85	352.47	$C_{20}H_{32}O_5$	0.477	Prostaglandin E2★	down	+++	+	/	/	+	+	-	Arachidonic acid metabolism
HMDB0008328	T3(ESI-)	756.563	9.38	758.06	$C_{42}H_{80}NO_8P$	2.534	PC(20:2(11Z,14Z)/14:0) △○●	down	+++	+	++	++	+	+	+++	Linoleic acid metabolism；Arachidonic acid metabolism；Glycerophospholipid metabolism；alpha-Linolenic acid metabolism
HMDB0010388	T3(ESI-)	516.317	8.25	517.64	$C_{26}H_{48}NO_7P$	1.903	LysoPC(18:3(9Z,12Z,15Z)) △○●	up	---	-	---	--	---	--	-	Linoleic acid metabolism；Arachidonic acid metabolism；Glycerophospholipid metabolism；alpha-Linolenic acid metabolism
HMDB0000394	T3(ESI-)	273.178	6.66	274.35	$C_{14}H_{26}O_5$	1.987	3-Hydroxytetradecanedioic acid△○●	down	+++	+++	+++	+++	+++	+++	+++	Biosynthesis of unsaturated fatty acids
HMDB0001644	T3(ESI-)	149.053	0.75	150.13	$C_5H_{10}O_5$	0.614	D-Xylulose○●	up	+++	+++	+++	+++	+++	+++	+++	Pentose and glucuronate interconversions
HMDB0011134	T3(ESI-)	319.235	9.14	320.47	$C_{20}H_{32}O_3$	0.286	5-HETE●	/	+	-	-	--	-	-	-	Arachidonic acid metabolism

HMDB0000122	T3(ESI-)	179.063	0.68	180.16	$C_6H_{12}O_6$	1.039	D-Glucose ○●	down	+++	+++	+++	+++	+++	+++	+++	Neomycin, kanamycin and gentamicin biosynthesis；Starch and sucrose metabolism；Galactose metabolism
HMDB0007913	T3(ESI-)	754.545	9.14	756.04	$C_{42}H_{78}NO_8P$	0.640	PC(14:1(9Z)/20:2(11Z,14Z)) ●	up	+++	/	-	-	--	-	-	Linoleic acid metabolism；Arachidonic acid metabolism；Glycerophospholipid metabolism；alpha-Linolenic acid metabolism
HMDB0013413	T3(ESI-)	741.565	9.12	742.06	$C_{42}H_{80}NO_7P$	0.140	PC(o-16:1(9Z)/18:2(9Z,12Z)) ●	down	+++	+	-	/	--	-	-	Linoleic acid metabolism；Arachidonic acid metabolism；Glycerophospholipid metabolism；alpha-Linolenic acid metabolism
HMDB0011213	T3(ESI-)	739.551	9.12	740.04	$C_{42}H_{78}NO_7P$	0.309	PC(P-16:0/18:3(9Z,12Z,15Z))	up	+++	/	-	/	-	-	/	Linoleic acid metabolism；Arachidonic acid metabolism；Glycerophospholipid metabolism；alpha-Linolenic acid metabolism
HMDB0008140	T3(ESI-)	779.546	9.14	780.07	$C_{44}H_{78}NO_8P$	0.219	PC(18:2(9Z,12Z)/18:3(6Z,9Z,12Z)) ○●	up	++	/	--	-	---	-	-	Linoleic acid metabolism；Arachidonic acid metabolism；Glycerophospholipid metabolism；alpha-Linolenic acid metabolism
HMDB0010381	T3(ESI-)	480.317	8.84	481.32	$C_{23}H_{48}NO_7P$	0.871	LysoPC(15:0) △○●	up	/	-	--	---	--	-	/	Linoleic acid metabolism；Arachidonic acid metabolism；Glycerophospholipid metabolism；alpha-Linolenic acid metabolism
HMDB0008175	T3(ESI-)	811.605	9.6	812.15	$C_{46}H_{86}NO_8P$	0.515	PC(18:3(6Z,9Z,12Z)/20:0) ●	up	+++	-	-	-	---	-	--	Linoleic acid metabolism；Arachidonic acid metabolism；Glycerophospholipid metabolism；

																alpha-Linolenic acid metabolism
HMDB0010408	T3(ESI-)	505.353	9.25	505.67	$C_{26}H_{52}NO_6P$	1.224	LysoPC(P-18:1(9Z)) △	up	/	/	/	/	-	/	/	Linoleic acid metabolism；Arachidonic acid metabolism；Glycerophospholipid metabolism；alpha-Linolenic acid metabolism
HMDB0008139	T3(ESI-)	807.573	9.21	808.12	$C_{46}H_{82}NO_8P$	0.391	PC(18:2(9Z,12Z)/20:3(5Z,8Z,11Z)) ●	up	++	+	-	-	--	-	-	Linoleic acid metabolism；Arachidonic acid metabolism；Glycerophospholipid metabolism；alpha-Linolenic acid metabolism
HMDB0000267	T3(ESI-)	128.043	1.13	129.11	$C_5H_7NO_3$	0.361	5-Oxo-D-proline	up	/	+	+	/	+	+	++	D-Glutamine and D-glutamate metabolism
HMDB0004243	T3(ESI-)	336.23	8.24	336.46	$C_{20}H_{32}O_4$	0.204	12(S)-HPETE●	up	/	/	-	-	--	-	--	Arachidonic acid metabolism
HMDB0061864	T3(ESI-)	279.24	8.43	280.45	$C_{18}H_{32}O_2$	0.951	Linoleate	down	-	+	-	+	-	+	+	Linoleic acid metabolism；Biosynthesis of unsaturated fatty acids
HMDB0062652	T3(ESI-)	295.235	8.92	296.45	$C_{18}H_{32}O_3$	0.599	9(10)-EpOME	up	+	/	/	-	-	/	-	Linoleic acid metabolism
HMDB0000036	T3(ESI-)	514.292	6.45	515.7	$C_{26}H_{45}NO_7S$	0.017	Taurocholic acid	/	+	-	-	+	+	+	++	Taurine and hypotaurine metabolism；Primary bile acid biosynthesis
HMDB0000112	Hilic Z(ESI+)	104.102	1.23	145.16	$C_4H_9NO_2$	1.112	4-Aminobutanoate★○●	/	/	---	---	---	---	---	---	Butanoate metabolism；Arginine and proline metabolism；Alanine, aspartate and glutamate metabolism
HMDB0000517	Hilic Z(ESI+)	175.118	5.24	174.2	$C_6H_{14}N_4O_2$	1.744	L-Arginine★△	down	+	/	/	/	/	++	+	Arginine and proline metabolism；Aminoacyl-tRNA biosynthesis；Arginine biosynthesis
HMDB0000159	Hilic Z(ESI+)	166.082	1.44	165.19	$C_9H_{11}NO_2$	0.989	L-Phenylalanine★	down	-	+	+	-	-	-	---	Aminoacyl-tRNA biosynthesis；Phenylalanine, tyrosine and tryptophan biosynthesis；Phenylalanine metabolism

HMDB0000162	Hilic Z(ESI+)	116.064	1.98	115.13	$C_5H_9NO_2$	1.158	L-Proline★○●	down	/	---	---	---	-	+	-	Arginine and proline metabolism；<br>Aminoacyl-tRNA biosynthesis
HMDB0000210	Hilic Z(ESI-)	218.111	0.92	219.24	$C_9H_{17}NO_5$	0.120	Pantothenate★	/	+	-	/	/	/	+++	+++	Pantothenate and CoA biosynthesis
HMDB0000208	Hilic Z(ESI-)	145.105	5.66	146.1	$C_5H_6O_5$	0.105	2-Oxoglutarate★●	/	++	+	+	+	++	+	+	Butanoate metabolism；<br>D-Glutamine and D-glutamate metabolism；<br>Alanine, aspartate and glutamate metabolism；<br>Arginine biosynthesis；Citrate cycle (TCA cycle)
HMDB0000064	Hilic Z(ESI-)	130.058	1.33	131.13	$C_4H_9N_3O_2$	1.360	Creatine★△○●	down	++	+	++	+++	+++	+++	+++	Arginine and proline metabolism
HMDB0000725	Hilic Z(ESI-)	130.059	2.8	131.13	$C_5H_9NO_3$	2.895	4-Hydroxyproline★△○●	up	-	---	--	-	--	-	-	Arginine and proline metabolism

Table S5 Topological information of urine metabolites in various periods

Topological information	Day7	Day21	Day35
network centralization	0.266	0.205	0.527
Avg. number of neighbors	1.694	1.632	2.181
characteristic path length	1.486	1.429	1.747
network density	0.044	0.042	0.020
numbers of nodes	35	35	87
numbers of edges	27	27	87

Table S6 The importance of input nodes in modular network

	KEGG	Metabolites	Degree	Betweenness centrality
Day7 urine	C02946	4-Acetamidobutanoate	1	0
	C01019	6-Deoxy-L-galactose（L-Fucose）	2	1
	C06424	Tetradecanoic acid（Myristic acid）	2	1
	C00312	L-Xylulose	1	0
	C05298	2-Hydroxyestrone	4	1
	C00106	Uracil	4	1
	C03758	4-(2-Aminoethyl)-1,2-benzenediol（Dopamine）	11	1
	C05828	Methylimidazoleacetic acid	1	0
	C00763	D-Proline	1	0
Day21 urine	C02946	4-Acetamidobutanoate	1	0
	C05584	3-Methoxy-4-hydroxymandelate（4-Hydroxy-3-methoxymandelic acid）	5	1
	C01089	(R)-3-Hydroxybutanoate	1	0
	C00780	3-(2-Aminoethyl)-1H-indol-5-ol（Serotonin）	5	1
	C01586	Hippurate（2-Methylhippuric acid）	1	0
	C01595	Linoleate	8	1
	C05643	6-Hydroxymelatonin	2	1
	C00312	L-Xylulose	1	0
	C00864	Pantothenate	1	0
	C06424	Tetradecanoic acid（Myristic acid）	2	1
Day35 urine	C05476	Tetrahydrocorticosterone	4	1
	C01089	(R)-3-Hydroxybutanoate	1	0
	C06424	Tetradecanoic acid（Myristic acid）	2	1
	C00166	Phenylpyruvate	7	1
	C00532	L-Arabitol	2	0
	C05634	5-Hydroxyindoleacetaldehyde	5	0.1175
	C00037	Glycine	49	0.9918
	C02714	N-Acetylputrescine	2	1
	C05587	3-Methoxytyramine	3	1
	C02336	Fructose	3	1
	C00735	Cortisol	8	1
	C00519	Hypotaurine	1	0

	C00025	L-Glutamate	39	0.7721
	C00042	Succinate	11	0.5987
	C00062	L-Arginine	16	0.4916
	C00157	Phosphatidylcholine（PC(20:2(11Z,14Z)/14:0)）	23	0.9444
	C00170	5'-Methylthioadenosine	8	0.2390
	C00245	Taurine	10	1
Day36 serum	C00300	Creatine	2	0.0357
	C00315	Spermidine	18	0.9007
	C01157	trans-4-Hydroxy-L-proline	4	1
	C01530	Octadecanoic acid（Stearic acid）	1	0
	C02990	L-Palmitoylcarnitine	1	0
	C04230	1-Acyl-sn-glycero-3-phosphocholine（LysoPC(18:3(9Z,12Z,15Z))）	7	0.1209

Table S7 Oral bioavailability prediction of AdmetSAR2.0 web-survice

Compound	Structure	Formula	MW	F/%
arctiin	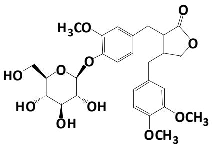	$C_{27}H_{34}O_{11}$	534.55	75
arctigenin	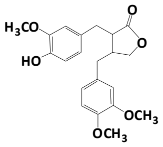	$C_{21}H_{24}O_6$	372.41	58
M1	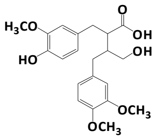	$C_{21}H_{26}O_7$	390.43	58
M2	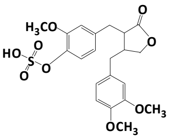	$C_{21}H_{24}O_9S$	452.47	54
M3	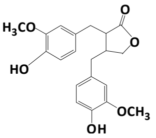	$C_{20}H_{22}O_6$	358.39	64
M4	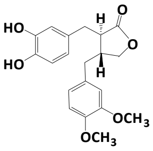	$C_{21}H_{24}O_5$	356.41	58
M5	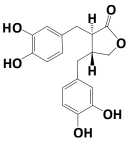	$C_{18}H_{18}O_6$	330.33	58

M6	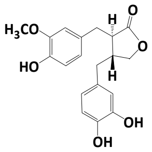	$C_{19}H_{20}O_6$	344.36	64
M7	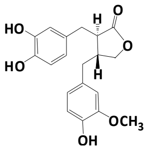	$C_{19}H_{20}O_6$	344.36	64
M8	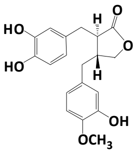	$C_{19}H_{20}O_6$	344.36	61
M9	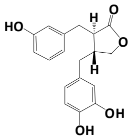	$C_{18}H_{18}O_5$	314.33	61
M10	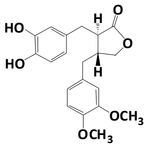	$C_{20}H_{22}O_6$	358.39	59
M11	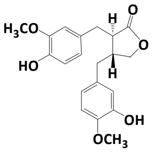	$C_{20}H_{22}O_6$	358.39	66
M12	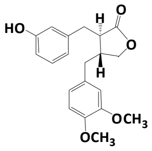	$C_{20}H_{22}O_5$	342.39	61
M13	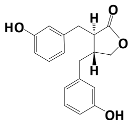	$C_{18}H_{18}O_4$	298.33	59
M14	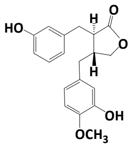	$C_{19}H_{20}O_5$	328.36	63

Table S8 XP Gscore of molecular docking studies

No.	Compound	Structure	XP GScore
1	arctigenin	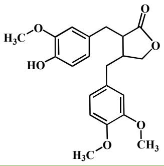	-5.69550

2	3′-desmethyl-arctigenin	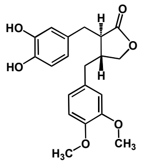	-6.40341
3	4',4"-dihydroxyl-enterolactone	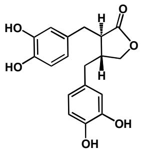	-9.20234
4	(2R,3R)-2-(3’-hydroxybenzyl)-3-(3”,4”-dihydroxybenzyl) butyrolactone	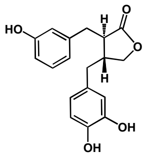	-8.42564
5	(-)-enterolactone	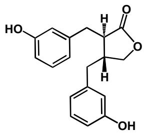	-7.89611
6	arctiin	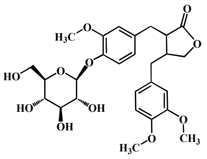	-7.34449
